# Multimodal Deep Learning with Attention-Based Fusion for Skin Cancer Diagnosis

**DOI:** 10.3390/bioengineering13050564

**Published:** 2026-05-16

**Authors:** Wiem Abdelbaki, Hend Alshaya, Inzamam Mashood Nasir, Sara Tehsin, Wided Bouchelligua

**Affiliations:** 1 College of Engineering and Technology, American University of the Middle East, Egaila 54200, Kuwait; wiem.abdelbaki@aum.edu.kw; 2 Applied College, Imam Mohammad Ibn Saud Islamic University (IMSIU), Riyadh 11432, Saudi Arabia; hialshaya@imamu.edu.sa; 3 Human-Environment-Technology (HET) Systems Centre, Mykolas Romeris University, 08303 Vilnius, Lithuania; inzamam.nasir@mruni.eu; 4 Faculty of Informatics, Kaunas University of Technology, 51368 Kaunas, Lithuania

**Keywords:** multimodal deep learning, skin cancer diagnosis, dermoscopic image analysis, clinical data fusion, attention mechanisms, cross-dataset generalization

## Abstract

The diagnosis of skin cancer remains a growing challenge because of its high variability as a result of the varying imaging conditions in clinical settings. This paper proposes a multimodal deep learning framework to address these challenges by combining the auxiliary clinical information with dermoscopic image features. This proposed architecture uses an attention-based feature encoder with a structured multimodal fusion approach to utilize the integrated feature representation across all channels. Evaluations of the proposed architecture were conducted across a range of benchmark datasets, including ISIC 2019, ISIC 2020, and HAM10000, using a unified experimental approach. This proposed model achieved accuracies of 90.5%, 88.7%, and 91.8% and AUCs of 95.8%, 94.6%, and 96.3%, respectively, on the selected datasets. For the baseline models, ResNet50 and EfficientNet-B4, our approach increased the AUC by 6.5% and the F1 score by 8.0%. Furthermore, across various datasets, the model achieved an AUC of 90.9%, proposing strong generalization. From the ablation analysis results, the attention and multimodal fusion mechanisms showed a 4.1% decrease in AUC when key components were removed, confirming their effectiveness. With 34.7 million parameters and an average of 19.3 Ms., the model has adequate intensity to deploy in a real clinical setting without affecting its performance. Additionally, the improvements to the model were statistically significant across all evaluation metrics (*p* = 0.01). The proposed multimodal framework demonstrates strong performance and robustness across multiple benchmark datasets.

## 1. Introduction

Skin cancer continues to be one of the most common and deadly forms of cancer. Melanoma, in particular, is very aggressive and has a high death rate if not found early. Because of the continuing rise of melanoma in the world, there is a need for fast and scalable methods to diagnose the disease. Current methods, such as biopsies and dermoscopic procedures, tend to be biased and rely heavily on the clinician’s skill. Recently, the field of automated skin lesion analysis has improved with the advent of deep learning. This has dramatically increased the rate of diagnosis and the ability to detect skin cancer in earlier stages [1]. This has also increased the development of computer-assisted diagnosis systems, which can improve clinicians’ ability to offer care, especially in areas with fewer resources.

Recent research has focused on convolutional neural networks (CNNs) for classifying skin lesions due to their ability to extract features from dermoscopic images and their spatial and textural characteristics. The classification ability of CNN-based models is further improved by leveraging transfer learning and data augmentation techniques [2]. Even with these advancements, CNN-based models have a disadvantage because they cannot model global and long-range contextual information, which is important for identifying lesions with similar visual characteristics. As a result, pure transformer and hybrid CNN-transformer models have been employed to address these issues and support representation learning via global attention [3]. These models consistently outperforms baseline models across the HAM10000 dataset in global attention-based representation learning.

Alongside architectural improvements, recent research seeks to build on previous efforts to address the intrinsic challenges of skin lesion datasets: class imbalance, sparse annotation, and domain disparity. For instance, ensemble learning and multi-scale feature extraction have been applied to enhance robustness and generalization in different lesion categories [4]. Moreover, in [5], it was found that dual-stage models that combine segmentation and classification outperform their peers in localization and classification performance due to the inclusion of lesion boundary models. Still, several approaches improve segmentation of lesion images, but their insufficient generalization across datasets and their sensitivity to imaging conditions remain major challenges in real-world clinical settings.

State-of-the-art research in multimodal deep learning has achieved important breakthroughs in skin cancer detection by integrating dermoscopic images with clinical metadata. This integration of data contributes to the depth of representation and, subsequently, to the accuracy of diagnosis. The current literature illustrates the performance of multimodal systems with clinical data in heterogeneous environments, contrasting them with unimodal systems [6,7,8]. Research shows that multimodal methods provide richer context to support decision-making and therefore outperform traditional image-only models [9,10]. Likewise, most recent multimodal frameworks using vision–language models have shown potential for addressing the intricate interplay between the visual and the semantic [11]. While many models boast high accuracy on particular datasets, recent studies show that their performance can decline significantly when evaluated across domains or when cross-dataset evaluations are used. Furthermore, the use of transfer learning and attention mechanisms has yielded some advances in interpretability and robustness; however, these approaches still face significant challenges in binary classification, including melanoma detection [12].

The contributions of this work are threefold. To begin with, a new multimodal deep learning framework is developed that combines dermoscopic image features with other clinical data to improve the diagnostic accuracy of skin cancer classifications. Using feature encoding and attention mechanisms, the model is designed to isolate and capture specific visual characteristics while leveraging clinical-structured attributes to add context. Second, a specific multimodal fusion approach is presented that supports effective representations of cross-modal interactions, thereby enabling enhanced representation learning and improved robustness across varying data distributions. Finally, the framework is thoroughly evaluated using various benchmarks, including HAM10000, ISIC 2019, and ISIC 2020. The framework continues to show improvements over strong baseline models and other frameworks, and, when subjected to multiple cross-dataset evaluations, it demonstrates strong generalizability. The framework also provides strong evidence for the validity of the proposed approach through thorough ablation studies and statistical significance analyses.

This paper is organized as follows: Section 2 reviews the literature. Section 3 proposes a methodology within the multimodal framework and the strategies of feature integration. Section 4 provides the experimental design and the evaluation protocol, followed by detailed results comprising the quantitative analyses, cross-dataset evaluation, and ablation studies. Lastly, in Section 5, the paper provides conclusions, principal findings, and the forecast of future lines of research.

## 2. Related Work

Diagnosis of skin cancer via automation has come a long way with deep learning models, especially convolutional neural networks, transformer learning, and multimodal representation learning, among other recent advancements. Most recent studies have aimed to improve classification, reduce class imbalance, and increase robustness by using heterogeneous data sets. However, the ability to generalize across a wide range of clinical datasets and settings remains a significant and unresolved issue.

Recent studies have evaluated the ability of multimodal infrastructures employing cross-attention, collaborative inference, and metadata integration to enhance both diagnosis and system scalability [13,14]. Furthermore, training transformers and foundation models on extensive dermatoscopic databases has been shown to deepen dermatoscopic understanding and the ability to apply clinically relevant demographic shifts across various groups [15,16]. Finally, several recent clinically oriented reviews have noted that, while various improvements have been reported, biases in datasets, non-interpretability, and the absence of regulatory frameworks continue to inhibit their application in the real world [7,17].

Recent research on the HAM10000 dataset has shown that hybrid models combining convolutional and transformer components can capture relevant local and global contextual features, thereby enhancing classification performance [18]. Likewise, design-efficiency models, such as quantization-aware neuromorphic models, have demonstrated that competitive accuracy can be achieved at lower computational expense [19]. Though these techniques provide evidence of the importance of both efficiency and innovative design for model construction, they often underestimate the extent of class imbalance in HAM10000. This phenomenon can result in biased learning towards the dominant lesion class. Therefore, the models’ performance on the less prevalent classes is significantly hindered.

Advancements in deep convolutional frameworks, in parallel scaling methods, and attention mechanisms have further strengthened discrimination power, resulting in high accuracy and AUC values [20]. On the other hand, the structural context of lesions is long-range relational covariates that cannot be modeled due to reliance on purely convolutional operations. In [21], multi-task learning strategies attempted to address this issue by integrating classification with the analysis of other clinical features, thereby enriching feature representation. This notwithstanding, the increased complexity of optimization and reliance on predefined clinical descriptors may impair adaptability to novel data distributions. Recently, improved generalization of multimodal contrastive learning methods has been examined to adjust and align visual representation and semantics [22]. This aforementioned method illustrates the use of relationships of cross-modal, and yet it is constrained by the scarcity of high-quality data sets and limited assessments across independent data sets. Riddled across the limitations stated collectively is the demand for data that addresses class imbalance and integrates multimodal data across datasets.

In recent studies, feature representation and fusion have been emphasized in deep learning models for binary skin cancer detection. Many models that use adaptive spatial feature fusion have been shown to perform well at detecting lesions at multiple scales. While these models appear to have good accuracy and AUC, they lack generalization to external datasets, most likely due to overfitting. A new class of models, the vision–language model, takes a new approach to generalization in dermatology by using large-scale multi-modal pre-training, which has been shown to be effective. Although these models have been shown to produce good results, they tend to perform poorly in specific areas, including overall model sensitivity and balanced accuracy, proposing they struggle to detect class imbalances and rare lesions. Likewise, dermatology-specific language-image models pre-trained on dermatology data have been shown to improve alignment between visual and semantic features. However, they are limited in practical application by their high data and computational resource requirements.

Multimodal knowledge-enhanced approaches attempt to integrate additional clinical data to bolster diagnostic performance [23]. These models help integrate visual and clinical reasoning; however, their complexity and effectiveness still depend on external knowledge sources. Moreover, large-scale biomedical vision–language models achieve competitive performance on weighted evaluation metrics [24], but differences between weighted and balanced metrics indicate class dominance, underscoring the need for more comprehensive evaluation methods.

The ISIC 2019 dataset has extensive skin lesion classification with multiple classes, due to its larger scale and greater variability than previous datasets. With the recent improvements to hierarchical concept-based modeling, the structured feature representation shows the benefits with the added strengths, but the improvements could be from the dataset itself, with the added improvements having less range from the different clinical variations, the most recent focal point being optimized representation learning across multiple ISIC 2019 datasets [22] and also focusing external and internal ISIC contrastive learning. These tend to be more recent, described as more refined data, and they also target provided training, making them less useful in their clinical world. Uncertainty-aware frameworks have been designed to help with the reliability improvements through more robust predictions in more challenging cases [25]. This possibility has typically been seen as a field example due to the added focus on computational complexity and scale.

Foundation models assessed on ISIC 2019 provide stronger evidence of multimodal pretraining potential for better generalization [26]. Similarly, knowledge-enhanced multimodal models, aided by extra contextual information, achieve remarkable performances [23]. However, both methods remain susceptible to domain shifts and may not adequately address the variability in extensive clinical datasets. Table 1 presents performance comparisons of the latest state-of-the-art techniques on the HAM10000, ISIC 2020, and ISIC 2019 datasets.

Although deep learning has greatly improved skin cancer diagnostics, current methods still have several shortcomings. First, most methods focus exclusively on dermoscopic images and ignore additional clinical data, which is critical for real-world scenarios. Furthermore, many models exhibit poor generalization across datasets and lose predictive power when the data domain changes. This is compounded by a lack of sophistication for capturing relevant data across different domains, which reduces the models’ ability to learn relevant features. Furthermore, current models tend to be overly general, failing to focus critically on specific diagnostic characteristics and resulting in poor feature learning for diagnostic features, particularly for challenging, rarely occurring lesion classes.

The proposed framework aims to advance the multimodal learning paradigm by integrating visual and clinical data to obtain finer, more identifiable representations. The attention-based feature encapsulation approach focuses the model on relevant aspects, thereby enhancing feature quality and interpretability. A specific fusion method enhances cross-modal interaction, enabling the model to leverage modality-specific complementarity. Also, the framework demonstrates significant improvement in robustness and generalizability across varying datasets and domains. This design helps the methodology to overcome the most critical limitations of the existing literature, offering a more reliable and comprehensive framework for skin cancer diagnostics.

## 3. Proposed Methodology

**Figure 1:**
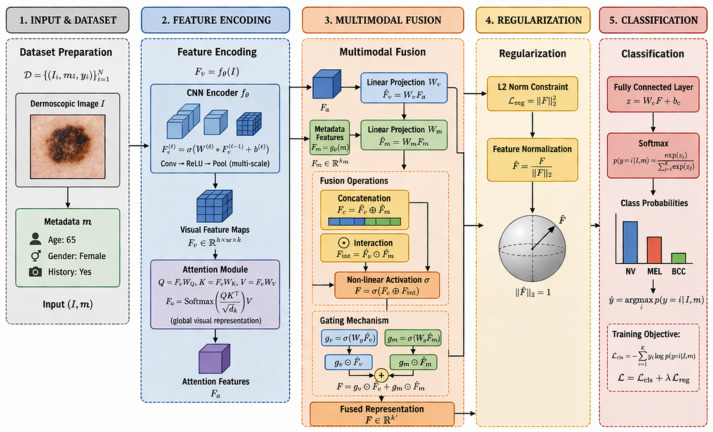
Overall architecture of the proposed multimodal framework for skin cancer diagnosis.

This paper presents a new multimodal deep learning framework for a skin cancer diagnostic system that uses dermoscopic images and other clinical information. Standard dermoscopic image-based deep learning systems can quantify lesion image disparities, but not the descriptors. The framework of this paper aims to bridge the gap and to use integrated visual, clinical, and fusion modules to obtain the most informative and diverse representations. The framework incorporates a holistic feature representation strategy, neural networks, and a multi-scale feature hierarchy. The framework implements other sophisticated techniques, such as an attention mechanism, to zero in on the most diagnostic features of an image. Furthermore, this framework utilizes a specialized clinical constructs module and cross-fusion to integrate clinical and visual representations. This work is an advancement towards deploying deep learning systems in practical clinical situations for skin cancer diagnostics. The refinements in features and the system’s ability to self-adjust are expected to facilitate greater practice generalization and capture more clinically relevant features in images. This work’s overall process, from data gathering and encoding to the fusion of multimodal features, the application of any designed safety features, and the predetermined classifications, is illustrated in Figure 1.

### 3.1. Problem Formulation

Let the input space be defined as X=(I,m), where $I\in R^{H\times W\times C}$ indicates a dermoscopic image, and $m\in R^{d}$ is the corresponding clinical metadata vector. The joint representation (I,m) combines visual and patient-specific contextual data, allowing for a more comprehensive characterization of the dermatological condition. The output space is defined as Y for multi-class classification, as discussed earlier. The goal of learning is to develop a parametric mapping function from the input space to label space while maintaining the discriminative gaps between classes. The model parameters Θ encompass all learnable components of the architecture, including the feature encoders, fusion components, and classification heads:

(1) f:X→Y

Let us define the predictive function as $f_{\Theta }$, mapping input samples (I,m) to predicted labels $\hat{y}$. During the prediction phase, a latent representation of the image and metadata (two modalities) is derived and subsequently integrated into one feature space. The prediction, $\hat{y}$, is essentially the result from the learned distribution concerning the most probable class. This approach addresses the synthesis of fundamental visual elements and high-level contextual relations, which are paramount for accurate diagnosis. We define the predictive function as follows:

(2) $$\hat{y}=f_{\Theta }\left(I,m\right)$$

The learning objective aims to place the problem in the context of risk minimization over the data distribution D. This includes determining the parameter set $\Theta^{*}$ that minimizes the expectation of the loss of the predicted labels as opposed to the true labels in the data with respect to the annotations. The loss function L(·) describes the difference between the predicted and actual labels and is most often formulated in terms of a divergence of two probability distributions. The expected risk is calculated from the joint distribution of the input variables and their associated labels, ensuring the model generalizes well to data beyond the training samples. The associated optimization problem can be expressed as follows:

(3) $$\min_{\Theta }\;E(I,m,y)∼D\left[L(f\Theta (I,m),y)\right]$$

To provide more details about the model’s behavior, including probabilities, the conditional distribution over the labels is defined. We have $p_{\Theta }\left(y|I,m\right)$ as the posterior probability for a given label *y* for the input (I,m). The posterior distribution is then maximized, leading to the interpretation that the function focuses on that label. This formulation helps incorporate uncertainty estimation while simultaneously providing the model with an informed framework for reasoning under uncertainty:

(4) $$\hat{y}=\arg\max_{y\in Y}\;p_{\Theta }\left(y|I,m\right)$$

When jointly modeling an image along with metadata, it is assumed that certain conditional dependencies exist between the modalities. This can be articulated by stating that the contribution of each modality is learned during the modeling/posterior distribution, which can be expressed as a function of both modalities. The model’s ability to capture complementary information while maintaining computational tractability is achieved through the factorization of the joint distribution:

(5) $$p_{\Theta }\left(y|I,m\right)=p_{\Theta }\left(y|F_{v},F_{m}\right)$$

where $F_{v}$ and $F_{m}$ are the latent representations from the image and from the metadata, respectively. With this formulation, we establish a link between the problem definition and the feature encoding process, aligning the problem with the methodology to be used in the subsequent steps. The merger of multiple modes of data and the probabilistic framework in the model increases the model’s ability to describe and understand intricate relationships that are hidden in the single-mode case:

(6) $$\left(F_{v},F_{m}\right)=\Psi_{\Theta }\left(I,m\right)$$

The function $\Psi_{\Theta }$ denotes the combined process in feature extraction, to which more detail will be provided in the following subsections. The overall formulation demonstrates the degree to which the coherency of the learning goal, the probabilistic modeling, and the feature representation are aligned, providing rigor to the proposed multimodal classification framework.

### 3.2. Dataset Preparation

This research employs three benchmark datasets: HAM10000, ISIC 2019, and ISIC 2020. A harmonized dataset preparation framework is developed to address heterogeneity in image resolution, acquisition conditions, granularity of image annotations, and distribution of image modalities. We denote the complete dataset as $D={\left(I_{i},m_{i},y_{i}\right)}_{i=1}^{N}$, with $I_{i}$ as the dermoscopic image, $m_{i}$ as the metadata, and $y_{i}$ as the label. This formulation combines all the datasets in post-processing, normalization, and label harmonization. The datasets are consistent in all but the statistical variables. The primary dataset spans a multi-class label space, whereas the primary datasets are binary-structured, thus requiring a common mapping for all:

(7) $$D={\left\{\left(I_{i},m_{i},y_{i}\right)\;|\;I_{i}\in R^{H\times W\times C},\;m_{i}\in R^{d},\;y_{i}\in Y\right\}}_{i=1}^{N}$$

There is significant variability in the input images’ spatial resolution and lighting, which can negatively affect the consistency of feature extraction. To tackle this problem, a preprocessing operator P(·) is introduced to standardize all images. This operator encompasses resizing, intensity normalization, and artifact reduction. If $I_{i}$ is the raw image, then the processed image ${\tilde{I}}_{i}$ can be expressed as

(8) $${\tilde{I}}_{i}=P\left(I_{i}\right)=R\left(N\left(A\left(I_{i}\right)\right)\right)$$

Here, A(·) denotes the removal of artifacts, N(·) denotes normalization of intensities, and R(·) denotes resizing. The removal of artifacts function lessens the effects of dermoscopy imaging noise caused by hair, ruler markings, and uneven lighting. The normalization function standardizes a range of pixel values to stabilize gradient-based optimization. By resizing the images, they all fall within the same spatial dimensions, facilitating effective batch processing and enabling consistent feature extraction. The normalization process is enhanced by applying a statistical transformation to the pixels to achieve a standardized distribution. Let μ and σ denote the mean and standard deviation of the pixels in the dataset, respectively. Then, the normalized image is given by

(9) $${\hat{I}}_{i}=\frac{{\tilde{I}}_{i}-\mu }{\sigma }$$

This transformation helps center and scale the distribution of inputs, thereby minimizing sensitivity to lighting and acquisition conditions. Moreover, the normalization maintains consistent gradient magnitudes, thereby facilitating faster convergence during training. Beyond global normalization, to sustain chromatic uniformity across the samples, channel-wise normalization is employed. Lastly, for robustness, the final component is the data augmentation operator T(·), which introduces variations in orientation, scale, and appearance. The augmented sample is formulated as

(10) $$I_{i}^{aug}=T\left({\hat{I}}_{i}\right)$$

where T(·) encompasses operations like rotation, flipping, scaling, and color perturbation. These operations increase the effective dataset size and enhance generalization by training the model on different variations of the same lesion. The augmentation strategy aims to balance the introduction of variability into the input space while preserving the dataset’s label space. For the primary dataset, the label space is Y=1,2,…,7, where each label corresponds to one of seven lesion categories. The label spaces of the auxiliary datasets are binary, so they require a mapping function to reconcile their differences. The transformation function is formulated from a clinical perspective, where the mapping categorizes malignant lesions as a single class and all other lesions as benign. This approach ensures that the classification remains clinically relevant while also enabling dataset interoperability:

(11) $$\phi \left(y\right)=\left\{\begin{array}{cc} 1, & y\in Y_{\mathrm{malignant}}\\ 0, & y\in Y_{\mathrm{benign}} \end{array}\right.$$

The adjusted label spaces become $Y^{\prime }=0,1$, where 1 shows malignant lesions and 0 shows benign lesions. Such a construction retains the model’s cross-class discrimination in a multi-class paradigm while also enabling it to tackle binary classification tasks. The hierarchical structure of label mappings aids the model’s comprehension of both micro- and macro-relational structures, thereby improving its overall generalization across datasets with differing annotation schemes:

(12) $$y_{i}^{\prime }=\phi \left(y_{i}\right)$$

To tackle the existing class imbalance in skin lesion datasets, both data augmentation and loss-level balancing techniques are used. Data augmentation focuses on underrepresented classes by applying transformations like rotation, flipping, and color perturbation. These techniques enhance the diversity of samples in the minority class and aid in representation learning. This method helps train the model on a more balanced distribution without modifying the original dataset structure.

The clinical metadata used in this study include patient-specific information, such as age, sex, and lesion location. These patient-specific features serve as contextual information that can enhance the model’s diagnostic utility when used alongside visual features. Before incorporation, all metadata features are normalized and encoded in a format suitable for digital embedding in a multimodal system. Multimodal learning presents challenges in dealing with missing metadata. Here, the missing clinical metadata is imputed using a basic imputation method. Age, a continuous variable, is represented by the average of the training set, while sex and lesion location, both categorical variables, are represented by a newly created “unknown” category. This approach allows incomplete data to be used for both training and evaluation while ensuring a consistent feature space.

### 3.3. Feature Encoding

The feature encoding module mainly relies on a CNN backbone to capture multi-scale spatial features within dermoscopic images. To boost representational efficiency, attention is applied over the feature maps. In this context, the attention module serves as a spatial self-attention mechanism that captures long-range dependencies across spatial areas within an image. Compared to transformer-based encoders, which use fully tokenized self-attention, the proposed mechanism combines deep attention and a CNN. Within the proposed mechanism, attention is applied to enhance local texture pattern capture, while a long-range CNN captures contextual relationships. This design guarantees the proposed mechanism focuses on region relevance while sustaining capture efficiency.

The feature encoding stage converts the processed dataset into structured latent representations that capture both the spatial and contextual elements of skin lesions. Let $I\in R^{H\times W\times C}$ be the processed dermoscopic image and $m\in R^{d}$ be the metadata vector. The goal is to develop a representation that captures visual patterns at different hierarchies and can be combined with metadata embeddings. Deep convolutional architectures have shown notable success in the medical image domain [29], and attention-based models excel at capturing long-range dependencies [30]. The encoding strategy combines both approaches to create a single representation that is insensitive to variations in lesions and acquisition conditions:

(13) $$F_{v}=f_{\theta }\left(I\right)$$

Each function $f_{\theta }\left(\cdot \right)$ denotes an individual hierarchical encoder with *L* transformation layers. Each successive layer captures increasingly more abstract features, allowing for a more fine-tuned refinement of the representation. Each layer *l* undergoes a transformation of a convolution operator followed by a non-linear function. This process allows the model to understand both low-level textural features and high-level semantic characteristics. Ref. [31] demonstrates that hierarchical representations improve the classification performance on dermatology imaging tasks. To enhance the encoder’s discriminative ability, the intermediate representation of layer *l* is recursively defined:

(14) $$F_{v}^{\left(l\right)}=\sigma \left(W^{\left(l\right)}*F_{v}^{\left(l-1\right)}+b^{\left(l\right)}\right)$$

Convolutional frameworks impose locality constraints that prevent the model from fully understanding global relationships across large spatial separations. This is applied to the encoded feature maps. Rather than focus on the immediate surrounding regions, attention mechanisms help capture relationships across distant regions that are critical in dermoscopic images, where the diagnostic features can be distributed [32]. With the attention mechanism, the feature maps are transformed into a representation that is contextually enhanced to capture the local and the global interactions:

(15) $$F_{a}=\mathrm{Attention}\left(F_{v}\right)$$

Attention mechanisms involve three projections that translate the feature space into distinct representations of queries, keys, and values. Such projections enable the calculation of similarity scores across spatial locations, which are then used to gauge the importance of each feature. This formulation has been shown to improve the quality of representations in medical image analysis [33]. The projections are specified as follows:

(16) $$Q=F_{v}W_{Q},\;K=F_{v}W_{K},\;V=F_{v}W_{V}$$

(17) $$F_{a}=\mathrm{Softmax}\left(\frac{QK^{⊤}}{\sqrt{d_{k}}}\right)V$$

Alongside visual encoding, a parameterized embedding function transforms the metadata vector *m* into a latent representation. The metadata contains contextual elements that images alone cannot capture, including demographic details and lesion location [34]. The embedding process maps metadata to a continuous feature space that integrates with visual representations, enabling efficient multimodal fusion at subsequent stages:

(18) $$F_{m}=g_{\psi }\left(m\right)$$

An embedding function can be described as a number of linear transformations followed by a non-linear activation function. Each transformation adds to the representation’s capacity to encode a greater number of complex relationships among the metadata components. This embodies a refinement process to ensure the metadata representation is sufficiently expressive to interact with the visual features:

(19) $$F_{m}^{\left(l\right)}=\sigma \left(W_{m}^{\left(l\right)}F_{m}^{\left(l-1\right)}+b_{m}^{\left(l\right)}\right)$$

**Lemma** **1.** *Let $F_{v}$ represent the visual feature representation derived from the encoder, and let $F_{a}$ represent the attention-enhanced representation. When the attention weights are normalized, the transformation of $F_{v}$ to $F_{a}$ preserves the boundedness of the feature space.*

**Proof.**  The softmax function determines the attention weights, and the softmax output, by definition, provides a probability distribution across the spatial dimensions. Hence, the weights are non-negative and sum to one. Let the attention weights be denoted as $\alpha_{ij}$, with *i* and *j* signifying positional indices. Consequently, $\alpha_{ij}\geq 0$, and $\sum_{j}\alpha_{ij}=1$. Therefore, the output representation at position *i* is one of the value vectors, expressed as a weighted sum:

(20) $$F_{a}^{\left(i\right)}=\sum_{j}\alpha_{ij}V^{\left(j\right)}$$

  □

**Figure 2:**
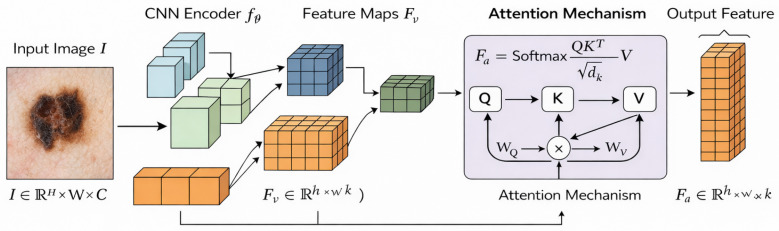
Feature encoding architecture of the proposed model.

Since $F_{a}^{\left(i\right)}$ is a convex combination of value vectors, its value will also be constrained by the maximum magnitude of an input feature. Therefore, if $F_{v}$ is constrained, $F_{a}$ will also be constrained. Thus, this bounding feature ensures numerical stability and prevents feature explosion, both of which are critical in deep architectures. Therefore, the feature encoding process creates a structured representation that captures and integrates the hierarchical convolutional features, global attention features, and contextual metadata embeddings. This integrated representation preserves the spatial and contextual information and provides a solid base for multi-level modality fusion, classification, and layered feature extraction. The model’s convolutional encoding, attention refinement, and metadata embedding enable it to learn a deep, sophisticated representation to address challenging, complex diagnostic problems. The process of feature encoding through hierarchical convolutional extraction and attention refinement is outlined in Figure 2.

### 3.4. Multimodal Fusion

The proposed approach differentiates itself from traditional early or late fusion techniques. Early fusion typically involves concatenating raw or low-level cross-modal features. This process can yield detrimental effects of representation potency due to the heterogeneity of the represented modalities. Late fusion does not delve into the granularity of cross-modal interactivity, as it combines separate predictions from each modality and performs post-modality-wise assessment. The proposed approach falls within the late fusion framework, where each modality is first encoded autonomously and then combined into a unified latent space. Compared to traditional concatenation-based fusion, the proposed approach uses a joint space for both addition and multiplication to delineate higher-order cross-modal interactions. In addition, unlike most cross-attention frameworks, the proposed approach employs a gated fusion mechanism as a “soft” modality selector. This mechanism is aimed at perhaps reducing cross-modal structural and representational complexity and increasing the model’s efficiency, enabling it to render an interactivity-neutral or structurally adequate output.

The fusion stage combines the encoded visual representation with the metadata, embedding it into a singular latent space conducive to unified reasoning. For this, let $R^{h\times w\times k}$ be the space of attention-enhanced visual representation, say $F_{a}$, acquired from feature encoding, and let $F_{m}\in R^{k_{m}}$ be metadata embedding. We aim to formulate a fusion operator that preserves specific modality characteristics while enabling cross-modal collaboration. Multimodal learning frameworks show that simpler forms of cross-interactions, such as concatenation-based fusion, do not capture the desired complex inter-modality dependencies, thereby requiring more sophisticated systematic projection and interaction [35]. Thus, the first step of the fusion process entails projecting both modalities to a unified latent space, ensuring that their representations are compatible in both dimension and semantics:

(21) $${\tilde{F}}_{v}=W_{v}F_{a},\;{\tilde{F}}_{m}=W_{m}F_{m}$$

In this case, $W_{v}\in R^{k^{\prime }\times k}$ and $W_{m}\in R^{k^{\prime }\times k_{m}}$ are the learnable projection matrices and $k^{\prime }$ is the dimensionality of the common latent space. The projection ensures the embedding of visual and metadata features in a unified coordinate space, enabling them to interact meaningfully. The transformation also helps regulate the scale and distribution of the features, which is a prerequisite for stable optimization. The projected representations ${\tilde{F}}_{v}$ and ${\tilde{F}}_{m}$ are then merged through a fusion operator to capture inter-modal dependencies:

(22) $$F=\sigma ({\tilde{F}}_{v}⊕{\tilde{F}}_{m})$$

While non-linear activation σ(·) captures interaction effects across dimensions, the concatenation operator ⊕ captures modality-specific features. This overview captures the model’s capacity to learn a composite representation that encompasses spatial structures and contextual features. However, the means of direct concatenation as a function lack the means to model fine-grained modality interactions. To counter this, an interaction term capturing multiplicative relationships among projected features is added. This interaction, by enabling the model to encapsulate dependence structures that additive ones fail to, augments the expressiveness of the fused representation:

(23) $$F_{int}={\tilde{F}}_{v}⊙{\tilde{F}}_{m}$$

where ⊙ signifies the point-wise multiplication operator. The interaction term highlights areas of dual-modal activity, strengthening the relevant features. The last fused representation is the sum of the interaction term and the concatenation-based representation, yielding a more expressive latent space:

(24) $$F=\sigma ({\tilde{F}}_{v}⊕\tilde{F}m⊕Fint)$$

To continue optimizing the fusion procedure, a gating mechanism is proposed to adaptively control the degree of contribution from each modality. The relevance of projected features determines the degree of gating and thus enables the adaptive adjustment of the importance of each modality. The gating coefficients for visual and metadata features are denoted as $g_{v}$ and $g_{m}$, respectively. These are derived from the projected representations, allowing the model to capture context-based weighting for each modality:

(25) $$g_{v}=\sigma \left(W_{g}{\tilde{F}}_{v}\right),\;g_{m}=\sigma \left(W_{g}{\tilde{F}}_{m}\right)$$

where $W_{g}$ is a learnable parameter matrix. The gated fusion representation is then defined as

(26) $$F=g_{v}⊙{\tilde{F}}_{v}+g_{m}⊙{\tilde{F}}_{m}$$

This formulation allows for the model to diminish the negative impact of irrelevant attributes while focusing on the more relevant differentiating attributes in each modality. The gating mechanism is especially useful when the quality of the accompanying metadata is inconsistent or when the visual attributes are overly dominant in the decision-making process.

**Lemma** **2.** *Let F be the representation obtained after the gated multimodal fusion. When the gating coefficients are such that $0\leq g_{v}\leq 1$ and $0\leq g_{m}\leq 1$, it follows that the fused representation is limited by the magnitudes of the projected features.*

**Proof.**  The fused representation can be understood as a weighted average of the projected features. Because the gating coefficients are produced by a sigmoid function, they are constrained to the range [0,1]. We impose the limits $C_{v}$ and $C_{m}$ on the norms of the projected features, i.e., $|{\tilde{F}}_{v}|\leq C_{v}$ and $|{\tilde{F}}_{m}|\leq C_{m}$. We have the following for the fused representation:

(27) $$\left|F|\leq \right|g_{v}⊙{\tilde{F}}_{v}|+|g_{m}⊙{\tilde{F}}_{m}|$$

  □

**Figure 3:**
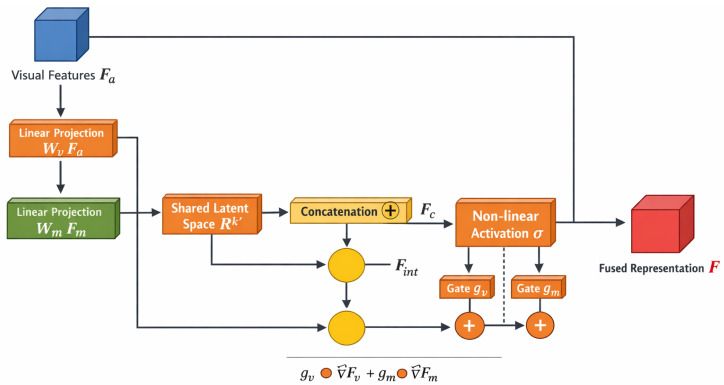
Multimodal fusion mechanism of the proposed framework.

Since $g_{v}$ and $g_{m}$ are bounded by 1, it follows that $|g_{v}⊙{\tilde{F}}_{v}\left|\;\leq \;\right|{\tilde{F}}_{v}|$ and $|g_{m}⊙{\tilde{F}}_{m}\left|\;\leq \;\right|{\tilde{F}}_{m}|$. Therefore,

(28) $$\left|F\right|\;\leq \;C_{v}+C_{m}$$

which shows that the fused representation stays within certain limits. This characteristic promotes numerical stability and prevents the magnitude of features from increasing without bound during training. To define the fusion process more clearly, an algorithmic description has been created that outlines the steps and the order of the operations involved in building the fused representation:

(29) $$F=F(F_{a},F_{m};\Theta_{f})$$

where F represents the fusion operator based on $\Theta_{f}$. The algorithm’s steps include projecting the two modalities, calculating interaction terms, implementing gating mechanisms, and creating the final fused representation. This process stabilizes and structurally processes the fusion of heterogeneous information sources and their effective integration. Consistent with the classification problem and the feature encoding, the representation *F* serves as the input to this stage. Figure 3 illustrates the multimodal fusion process that includes interaction modeling, shared projection, and gated aggregation.

### 3.5. Representation Regularization

The multimodal fusion stage results in a representation denoted as $F\in R^{k^{\prime }}$, where $k^{\prime }$ is the dimensionality of the common latent space. To stabilize the representation space, it is regularized to improve robustness. Regularization, in most cases, aims to keep the learned features stable within a limit and to make them invariant to changes in the input data. Regularization, in most cases, is how deep learning handles overfitting and improves generalization performance [36]. In the case of multimodal data, regularization maintains balance across modalities and prevents any single modality from dominating:

(30) $$L_{reg}={\left|F\right|}_{2}^{2}$$

The notation Lreg refers to the fused feature vector’s squared Euclidean norm. This constraint favors features of smaller magnitude and promotes a more space-compact representation in the latent space. Let $F=(f_{1},f_{2},\ldots ,fk^{\prime })$, then the regularization term can be expressed as a sum of squared elements. This method guarantees that the punishment is equitably assigned throughout all feature dimensions, and circumvents the possibility that one dimension overshadows the others. This restriction, by constraining the size of the gradients, impacts the size of the fluctuations, yielding smoother training and improved stability of optimization:

(31) $$\left|F\right|2^{2}=\sum {i=1}^{k^{\prime }}f_{i}^{2}$$

In addition to controlling magnitude, normalization makes the merged representation scale-invariant. Normalization can be thought of as projecting a feature vector onto a unit hypersphere so that all representations have the same norm. In multimodal learning, different modalities of data likely exhibit different feature scales; this type of transformation becomes critical. By funneling the model’s focus to directional data rather than magnitudes, the unit norm protects against changes in input magnitudes and in the distributions of the critical underlying metadata:

(32) $$\hat{F}=\frac{F}{{\left|F\right|}_{2}}$$

The representation $\hat{F}$ is constrained to the surface of the unit sphere in $R^{k^{\prime }}$. This constraint imposes a geometric structure on the feature space that enables systematic comparison of samples. In addition, the normalization preserves and enhances numerical stability, especially when combined with gradient-based optimization, and reduces the feature space’s sensitivity to noise. Moreover, in [37], the effect of normalization on the convergence properties of deep neural networks is discussed and attributed to its role in maintaining consistent activation levels across layers. When the batch mean of the fused representation is denoted by $\mu_{F}$, the variance regularization term introduced to improve the stability of the features encourages the dispersion of the features around the mean to minimize the variability and improve the homogeneity across the samples:

(33) $$L_{var}={\left|F-\mu_{F}\right|}_{2}^{2}$$

In the presence of $L_{var}$, the learned representations are constrained from exhibiting excessive variance, which could result in unstable predictions. This constraint is especially relevant in medical imaging, where a reliable diagnosis often depends on the consistent representation of the same pathological entities.

**Lemma** **3.** *Let $\hat{F}$ represent the normalized fused representation. Since $|\hat{F}{|}_{2}=1$ for all samples, the representation is on a compact manifold.*

**Proof.**  By definition, the normalized representation is given by $\hat{F}=F/{\left|F\right|}_{2}$. The norm of $\hat{F}$ is therefore:

(34) $$|\hat{F}{|}_{2}={\left|\frac{F}{{\left|F\right|}_{2}}\right|}_{2}$$

  □

Using the homogeneity property of norms, it follows that

(35) $$|\hat{F}{|}_{2}=\frac{{\left|F\right|}_{2}}{{\left|F\right|}_{2}}=1$$

Therefore, all normalized representations are located on the unit hypersphere, which, being closed and bounded, qualifies it as a compact set. The stability of the optimization process depends upon the feature space being prevented from divergence, indicating that the feature space is being restricted to a finite area. The total regularization objective merges a magnitude, normalization, and variance to attain a feature representation that is stable and robust. The complete formulation of the regularization loss is expressed as follows:

(36) $$L{total}^{reg}=Lreg+\lambda_{1}L_{var}$$

where $\lambda_{1}$ is a weighting factor that balances the contribution of the variance regularization term. This way, the formulation ensures that the fused representation is bounded, normalized, and consistent across samples, offering a firm basis for the subsequent classification stage.

### 3.6. Classification Objective

In the classification stage, the goal is to map the regularized and fused representations to the relevant prediction space as specified in the problem definition. Let $F\in R^{k^{\prime }}$ be the fused representation. Let $\hat{F}$ denote the normalized representation from the regularization stage. The classification head is defined by parameters ω and is described as a set of linear and non-linear mappings that transform the latent representation into the logit space. This mapping maintains the learned structural relationships from the feature encoding and multimodal fusion, and the space allows for discriminative separation for the different classes. Because of their ability to define complex decision surfaces, deep classifiers that operate in the space of the learned features have been found to perform particularly well for tasks involving the classification of medical images [38]:

(37) $$\hat{y}=h_{\omega }\left(F\right)$$

The classification head is viewed as a linear transformation and an activation function. Let $z\in R^{K}$ be the logit vector and *K* be the number of classes. The described transformation can be viewed as a projection of the fused representation into logit space using a weight matrix and a bias vector. This projection gives a score to each class, reflecting the possibility of each class given the input representation:

(38) $$z=W_{c}F+b_{c}$$

In this context, $W_{c}\in R^{K\times k^{\prime }}$ pertains to the classification weight matrix, with $b_{c}\in R^{K}$ relating to the bias vector. The model produces logits, which indicate the model’s confidence estimates for each class. These logits are then transformed into class probabilities after applying a normalization process. For formulating the loss function and optimizing through the gradient descent methodology, it is essential to grasp the logits as probabilities:

(39) $$p\left(y=i|I\right)=\frac{\exp(z_{i})}{\sum_{j=1}^{K}\exp\left(z_{j}\right)}$$

The posterior probability of class *i* given the input image and its associated features is given by the distribution of p(y=i|I). The softmax function normalizes the probability distribution to a valid categorical distribution, ensuring that the probabilities sum to one, and also creates a competitive environment among the class distributions by emphasizing the most relevant class and suppressing the probabilities of all other classes. This is a reliable and consistent method for multi-class classification, and further, softmax is probabilistically aligned to the fundamentals of probability [39]. The difference between the actual labels and the predicted probabilities is described using a loss function, and we represent the true label as a one-hot vector, y∈Y. The classification loss function for this problem is cross-entropy, which is the most popular due to its solid theoretical foundations and the extensive literature [40]:

(40) $$Lcls=-\sum {i=1}^{K}y_{i}\log p\left(y=i|I\right)$$

Cross-entropy loss discourages incorrect predictions by greatly increasing the loss when the model assigns low probabilities to the true class. This motivates the model to reliably make correct predictions. The loss function is also differentiable, making it easier to optimize using gradient-based algorithms. The loss gradients are backpropagated to the classification head and to every preceding part of the model, such as the feature encoding and the fusion modules, and used to update the features. A more weighted class-loss function can also be used to address class imbalance and improve confidence. In this case, let $w_{i}$ be the weight associated with class *i* and its significance. The weighted loss becomes more significant during training for the underrepresented result:

(41) $$Lwcls=-\sum {i=1}^{K}w_{i}y_{i}\log p\left(y=i|I\right)$$

The overall optimization goal integrates the classification loss and the earlier defined regularization term. This combined goal balances high classification accuracy with stable, well-structured feature representations. The classification and regularization balance is determined by a hyperparameter that governs the overall contribution of the regularization term:

(42) L=Lcls+λLreg

**Lemma** **4.** *Let Lcls represent the cross-entropy loss defined over the softmax output. Then it holds that Lcls is convex with respect to the logits z.*

**Proof.**  The cross-entropy loss can be decomposed into a negative log-likelihood and a softmax function. The softmax function is differentiable and transforms logits into probabilities. Negative log-likelihood, in terms of a given probability distribution, is a convex function. Because the composition of a convex function and an affine function preserves convexity, the loss function is convex in terms of the logits. Thus, it follows that $L_{cls}$ is convex in *z*, which implies that there is a unique global minimum for optimization with respect to the logits.   □

Therefore, the classification aim provides a systematic structure for representing multimodal data to yield a predictive overview. Reliability and accuracy are brought to the models thanks to the combination of probabilistic modeling, a weighted-loss formulation, and regularization. The inter working of the components ensures to provide a complete pathway from both the feature encoding and fusion stages to the stages of decision making, and also to ensure that effective learning occurs from the various data sets.

## 4. Results and Discussion

The proposed multimodal framework’s performance has been evaluated on various benchmark datasets, including ISIC 2019, ISIC 2020, and HAM10000. From these datasets and analyses, conclusions regarding the framework’s classification performance, model robustness to domain shifts, and specific architectural analyses are possible. Normally, the proposed framework assesses the best-performing baseline models, since the competitive baseline models analysis is performed in parallel to the framework. A set of detailed class-wise analyses, generalization results for framework performance, and ablation results collectively demonstrate the framework’s performance. Statistically, the framework’s performance results indicate that it is appropriate for real-life diagnostic practice overall.

### 4.1. Experimental Setup and Evaluation Protocol

The diverse nature of lesion types and acquisition circumstances has motivated the analysis of the proposed framework on the ISIC 2019, ISIC 2020, and HAM10000 benchmark skin cancer datasets. All images were resized and normalized to a single resolution prior to training, and data augmentation was applied to improve generalization. This included random horizontal and vertical flipping, random rotation, and jittering of the images, and then standard practices were applied to partition the data into training, validation, and testing subsets. Training was performed with the Adam learning rate scheduler, and validation loss was used to implement early stopping to prevent overfitting. Evaluations were performed on multiple metrics, including Accuracy, Precision, Recall, F1-score, and the Area Under the ROC Curve (AUC). To maintain experimental control, the analysis was conducted three times with different random seeds to ensure reproducibility and statistical reliability.

**Figure 4:**
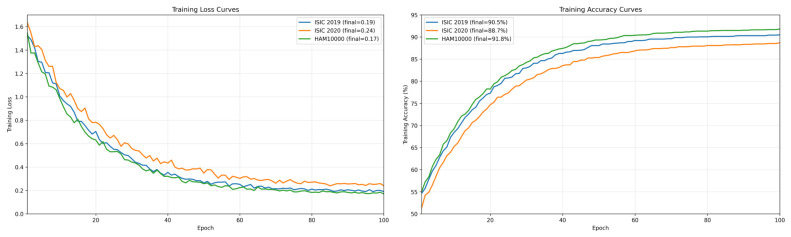
Training dynamics of the proposed model on ISIC 2019, ISIC 2020, and HAM10000.

The training configuration for each dataset is summarized uniformly in Table 2. This means that the same hyperparameters have been applied to all three datasets, ISIC 2019, ISIC 2020, and HAM10000, so that performance can be compared across datasets without the need to fine-tune hyperparameters specific to a dataset. The chosen batch size is a trade-off between the desired stability of the gradients and the need for computational efficiency. The learning rate is set to a small range and is adjusted using a cosine scheduler. This allows for smooth changes to the learning rate, aiding the final convergence. Dropout and weight decay regularization have been used to mitigate overfitting in the presence of class imbalance. The data augmentation parameters have been set to realistic alterations that dermoscopic images may encounter across the multiple settings in which they might have been taken. This further develops the model learning across different settings. To enhance the model’s generalization, training is stopped early to prevent training from occurring after the model has converged. The behavior optimization across datasets, in terms of minimizing loss and improving accuracy, is depicted in Figure 4.

In addition to the hyperparameters in Table 2, additional tools of the training process for the hyperparameters are employed, including early stopping for the validation loss, employing the patience strategy for observing early stopping of the validation loss during training, and implementing decay of the learning rate as a cosine function. To compare the models and ensure reproducibility of results, all models are trained using identical training configurations for all datasets. The training process uses the same methods for preprocessing and data augmentation, and the same strategies for iterations. To ensure reproducibility and maintain experiment reliability, the dataset was divided into three subsets: training, validation, and test. This was done through stratified sampling. Within this context, 70% of the dataset was divided into the training subset, 15% into the validation subset, and the remaining 15% into the test subset. Stratified sampling was applied at the patient level to prevent information leakage across subsets. Additionally, to ensure the consistency and reliability of the outcomes, the experiments were repeated three times with different random seeds. The results presented in this research are representative of the mean and standard deviation across the three repeated trials.

To make an accurate comparison among baseline models, ResNet50, EfficientNet-B4, and the variants inspired by the transformer style, all baseline models are subjected to the same experimental conditions. These conditions include identical data splits, identical preprocessing pipeline, identical augmentation, identical optimizer settings, identical learning rate schedule, and identical number of training epochs. No hyperparameter tuning for individual models is done outside of standard settings. No experimental bias is present, and differences in performance are assumed to be the objective.

### 4.2. Quantitative Performance

**Figure 5:**
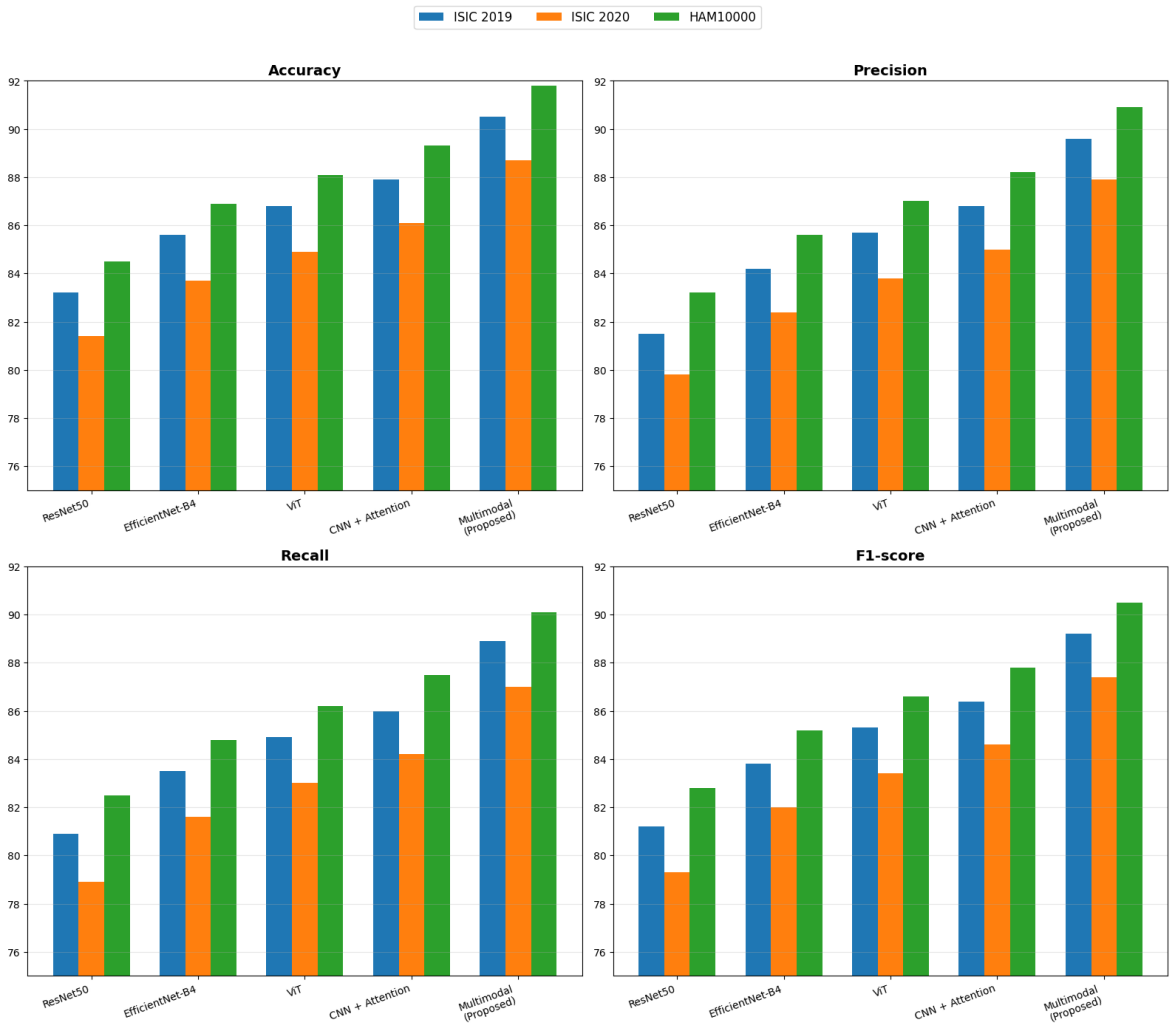
Quantitative performance comparison of baseline and proposed methods across ISIC 2019, ISIC 2020, and HAM10000.

The proposed framework demonstrates the ability to learn discriminative, generalizable representations across datasets despite their heterogeneity across multiple dimensions of complexity. As shown in Table 3, the proposed multimodal model on ISIC 2019 achieves 90.5 in accuracy, 89.6 in precision, 88.9 in recall, 89.2 in F1, and 95.8 in AUC, surpassing the most competitive CNN + Attention baseline by 2.6 in accuracy and 2.4 in AUC. These scores reflect a statistically significant enhancement in the ability to separate decision boundaries. In ISIC 2020, the proposed model remains the most competitive, with 88.7 in accuracy and 94.6 in AUC, as all models show a drop in performance due to the increased dataset variability. This shows an increased performance under difficult conditions. On HAM10000, the proposed model achieves 91.8 in accuracy and 96.3 in AUC, outperforming CNN + Attention (89.3 in accuracy and 94.1 in AUC). This indicates that the proposed model also has a better ability to detect and capture the fine-grained details of the lesions. In Figure 5, the performance metrics from multiple evaluations and datasets are demonstrated.

The dataset-wise summary provides a coherent overview of the proposed model’s behavior across datasets with varying characteristics and demonstrates its stability, high performance, and rapid learning. The model achieved 90.5 accuracy, 89.6 precision, 88.9 recall, 89.2 F1 score, and 95.8 AUC on ISIC 2019, as shown in Table 4, comparable to ISIC 2020 (88.7 Accuracy and 94.6 AUC), despite the dataset being more complex. The model achieved 91.8% accuracy and 96.3% AUC on HAM10000, demonstrating its ability to learn more complex, fine-grained lesion patterns in larger datasets. On the consolidated dataset, the model achieved a high performance of 90.9% accuracy, 90.0% precision, 89.3% recall, 89.6% F1 Score, and 95.9% AUC, demonstrating its ability to learn a heterogeneous dataset without performance degradation.

The effectiveness of the imbalance-handling strategy is evidenced by consistent performance across both dominant and minority classes, as shown in the class-wise evaluation. Even with skewed class distributions in datasets such as HAM10000, the model achieves competitive recall and AUC across all lesion types, demonstrating that the implemented balancing techniques effectively reduce bias toward the majority classes.

### 4.3. Class-Wise Performance Analysis

A class-wise breakdown in Table 5 provides an in-depth review of the proposed model’s ability to allocate its predictive power across different lesion types based on lesion frequency and visual complexity. In the table referenced above, the model demonstrates a sensitivity of 94.5 and a recall of 86.9 for melanoma lesions, and a sensitivity of 86.9 and an AUC of 94.5, allowing it to respond to clinically relevant malignant lesions despite their variability. The model, demonstrating its ability not only to identify the dominant characteristics of a given class but also to retain its separation from other class-related characteristics, scored an AUC of 96.8 and an accuracy of 92.4 for nevus lesions. The model achieved accuracies and AUC scores of 90.2 and 95.7 for basal cell carcinoma, and 91.1 and 95.9 for benign keratosis lesions, respectively, indicating consistent features across both lesion types. The model maintained accuracy and AUC above 87.9 and 88.8 for actinic keratosis and squamous cell carcinoma, respectively, indicating it performed effectively despite minor differences between the categories. The model achieved accuracies of 89.3 and 90.7, and AUCs above 94.8 for dermatofibroma and vascular lesions, respectively, confirming that it avoided the trap of favoring the higher-frequency lesion types.

The evaluation of the proposed model through the lens of the binary classification problem shows that it achieves balanced discrimination across both malign and benign categories, whilst also maintaining sensitivity to cases of clinical concern. As seen in Table 6, the model shows a strong ability to capture malignancy, achieving 87.2 accuracy, 86.5 precision, 85.6 recall, 86.0 F1 score, and 93.9 AUC for melanoma, which is impressive given the high intra-class heterogeneity and the presence of benign lesions. For benign cases, the model achieves 90.1% accuracy, 89.3% precision, 88.5% recall, 88.9% F1 score, and 95.2% AUC, indicating the model is consistent in correctly identifying non-malignant cases. Given the small difference in melanoma and benign performance, one could infer that the model’s decision boundary is neither too conservative nor too liberal toward the benign class, proposing high calibration.

**Figure 6:**
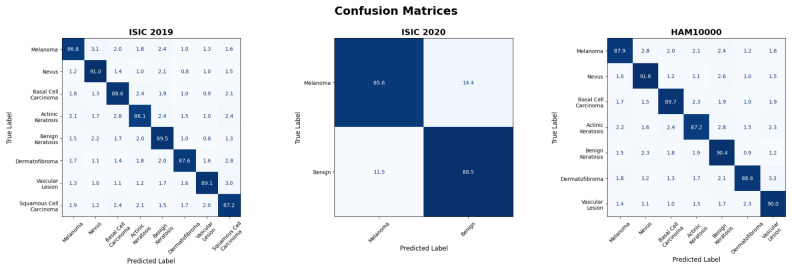
Confusion matrices of the proposed model on ISIC 2019, ISIC 2020, and HAM10000.

**Figure 7:**
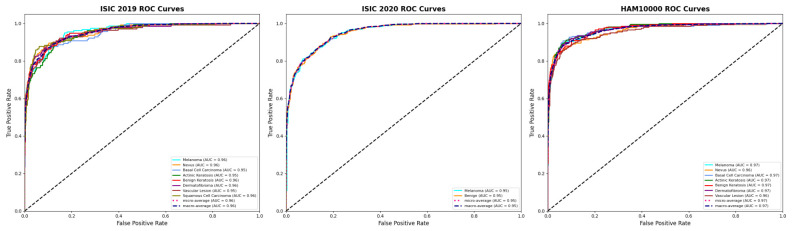
Receiver operating characteristic curves of the proposed model on ISIC 2019, ISIC 2020, and HAM10000.

The evaluation of HAM10000 on a class-by-class basis shows that the proposed model consistently performs well across lesion categories, even as visual complexity and sample distributions increase. Table 7 shows that the model for melanoma has an AUC of 95.6% and an accuracy of 89.5%, 88.7% precision, 87.9% recall, and an F1-score of 88.3%, demonstrating that the model can reliably recognize malignant patterns even when the patterns are difficult to identify. For the case of nevus, performance is even higher, with an accuracy of 93.1% and an AUC of 97.2%, demonstrating that the model can learn to represent the dominant classes while keeping them separate from the other classes. Stable performance is also shown on BCC and BK across malignancy and benign levels, with 91.3% accuracy and AUC of 96.4%, and 92.0% accuracy and AUC of 96.6%, respectively. In the case of Actinickeratoses, these few visual differences pose challenges, and with an AUC of 94.9% and an accuracy of 88.8%, it demonstrates consistent performance when finely differentiated. Furthermore, Erythematous dermatofibroma and vascular lesions show consistent performance with 90.2% and 91.6% accuracy, respectively, and AUCs of 95.7% and 96.3%, proposing that the majority of the classes negatively impact performance. The class-level performance across the datasets is further illustrated using the confusion matrices in Figure 6. The class-wise and aggregate discrimination ability of the model is further demonstrated by the ROC curves in Figure 7.

### 4.4. Cross-Dataset Generalization

The cross-dataset evaluation in this study is an external validation method; the model is evaluated across datasets that differ in distribution, acquisition conditions, and annotation schemes. This method ensures learned representations that generalize beyond a single dataset and across multiple, independent cohorts. This kind of validation is especially warranted in medical imaging settings, where inter-clinical center variability is considerable. Table 8 depicts the transfer from ISIC 2019 to ISIC 2020 and the proposed model’s ISIC 2020 AUC of 90.1 and ISIC 2019 82.0 F1 score, which are over the top-performing baseline CNN + Attention’s 87.2 AUC and 80.1 accuracy. This illustrates the proposed framework’s improvement in domain robustness. In contrast, across ISIC 2020 to ISIC 2019, the model was even more successful, with ISIC 2020 AUC at 90.8 and 84.2 accuracy, which improves upon the CNN + Attention, which had only 81.0 and 87.8, respectively, illustrating consistent bidirectional transferability. From ISIC 2019 to HAM10000 ISIC 2019, the model’s ISIC 2019 AUC of 91.7 and accuracy of 85.6 surpass the baseline’s ISIC 2019 AUC of 88.9 and accuracy of 82.2, demonstrating the model’s generalization from larger, more varied datasets. In the ISIC 2019 to HAM10000 transfer, the model achieved an ISIC 2019 AUC of 91.0 and an accuracy of 84.9, compared to the top-performing baseline’s AUC of 88.2 and an accuracy of 81.6, reinforcing the model’s stable adaptation across disparate datasets and class distributions.

The combined performance across datasets offers insights into the model’s consistency across multiple transfer scenarios and stresses its predictive performance under domain shifts. As noted, in Table 9, the model’s performance in the transfer from ISIC 2019 to ISIC 2020 shows 83.4 accuracy, 82.5 precision, 81.6 recall, 82.0 F1 score, and 90.1 AUC, from which there is an observed improvement in the reverse direction to 84.2 accuracy, and 90.8 AUC showing consistent bidirectional generalization. The most significant performance improvement was observed in the transfer from HAM10000 to ISIC 2019, achieving 85.6% accuracy and 91.7% AUC, indicating an impressive transfer driven by a more ample and varied training distribution. This aligns with the transfer from ISIC 2019 to HAM10000, which achieved 84.9% accuracy and 91.0% AUC, indicating consistent and robust adaptation across datasets with varied class compositions. The summarized performance across all scenarios shows that the model’s performance did not drop significantly due to the domain shift. The model achieved an average performance of 84.5% accuracy, 83.6% precision, 82.8% F1 score, and 90.9% AUC.

### 4.5. Ablation Analysis

The effects of the modules, individually and collectively, on the model’s overall behavior, representations, and predictive capabilities can be evaluated through an ablation study. The Table 10 shows the results of the full model, which achieves a 90.5 accuracy, 89.6 precision, 88.9 recall, 89.2 F1-score, and 95.8 AUC. This sets the upper bound of its performance. When the attention encoder is removed, the performance drops to 88.2 accuracy and 93.6 AUC. This shows that the attention mechanism contributes to improvements in feature discrimination and the contextual model. Removing the fusion module results in an even larger drop, with performance falling to 86.4% accuracy and 92.1% AUC, indicating that cross-modal integration is the most important contributor to the performance improvements. Excluding the clinical features yields 87.1% accuracy and 92.9% AUC, indicating that the non-imaging support helps refine the decision boundaries. The worst performance is demonstrated by the CNN-only configuration, which shows 85.2 accuracy and 90.8% AUC. This shows the ineffective nature of unimodal feature extraction.

The ISIC 2020 ablation results support the individual contributions of each architectural element in the more complex, higher intra-class overlap binary classification. Table 11 shows the strongest overall performance with the full model at 88.7 accuracy, 87.9 precision, 87.0 recall, 87.4 F1-score, and 94.6 AUC. Removing the attention encoder yields 86.3% accuracy and 92.4% AUC, underscoring the importance of refining contextual features to distinguish melanoma from benign cases. The greatest degradation occurs without a fusion module, with 84.8% accuracy and 91.0% AUC, indicating decreased performance and reiterating the importance of cross-modal interaction for acquiring essential complementary information for melanoma detection. The absence of clinical features achieves 85.5% accuracy and 91.7% AUC, further confirming the importance of auxiliary metadata for consistent decisions. The configuration with only a CNN achieves the highest domain complexity, with 83.7 accuracy and 89.8 AUC, further reiterating the limitations of unimodal representations.

The ablation study conducted on the HAM10000 dataset confirms the effect of the individual components on the overall model performance, using a larger, more heterogeneous dataset with more detailed class differences. The results for the full model presented in Table 12 show the best results with an accuracy of 91.8, a precision of 90.9, a recall of 90.1, an F1-score of 90.5, and an AUC of 96.3. When the attention encoder is removed, the results drop to 89.6% accuracy and 94.2% AUC, confirming that attention indeed improves the modeling of contextual features. The performance decline is even more pronounced when the fusion module is removed—the accuracy drops to 87.9 and the AUC to 92.8, confirming that the multimodal fusion is the main contributor to the performance improvement. When the clinical features are excluded, the model achieves 88.5% accuracy and an AUC of 93.5, indicating that additional context supports more consistent predictions. The configuration with only the CNN features has the worst performance, with 86.8% accuracy and 91.6% AUC, indicating that purely visual features lack sufficient predictive power.

The various methods of feature integration are significant for modeling performance, and the analysis highlights the importance of cross-modal interaction. As illustrated in Table 13, simple concatenation results in an accuracy of 88.1 and an AUC of 93.2, indicating that direct feature stacking closes minimal inter-modality gaps. An element-wise summation reduces performance to 87.5% accuracy and an AUC of 92.7%, implying that simple summation, in the absence of learnable weights, results in the loss of critical information. Gated fusion boosts performance to 89.2 accuracy and 94.1 AUC, demonstrating that control superpositions of learnable weights are important for balanced feature contributions. Gated fusion is surpassed by the proposed attention-based fusion, which achieves 90.5 accuracy, 89.6 precision, 88.9 recall, 89.2 F1 score, and 95.8 AUC, which is 1.3 greater in accuracy and 1.7 greater in AUC. This means that the attention mechanism is enabled and flexible for cross-modality feature alignment, and that the model is capable of dynamically balancing important features and silencing unimportant or noisy ones.

Clinical-feature integration analysis shows that patient metadata improves model predictions when combined with images. The image-only configuration in Table 14 achieves 87.1 accuracy and 92.9 AUC, indicating strong performance on visual-only data. While clinical data alone shows 78.4% accuracy and an AUC of 85.3%, clinical metadata lacks distinct information and, on its own, is insufficient for reliable classification. When both image and clinical data are concatenated, performance improves to 88.4% accuracy and 93.8% AUC, indicating that adding clinical metadata improves performance. Our enhanced integration method improves performance to 90.5 accuracy, 89.6 precision, 88.9 recall, 89.2 F1-score, and 95.8 AUC, an increase of 2.1 accuracy and 2.0 AUC over concatenation. The results show that careful, structured interaction among modalities is more effective for capturing the predictive value of the data to support the model in optimizing its predictions, leveraging the data’s visual patterns and the underlying clinical information that provides explanatory context.

**Figure 8:**
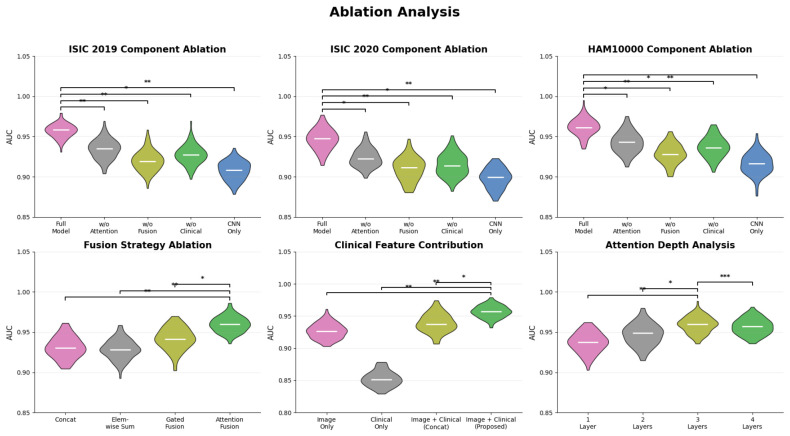
Visual summaries from the ablation analyses. The violin plots illustrate the distribution of the AUC score achieved through component-wise ablation on ISIC 2019, ISIC 2020, and HAM10000, alongside the fusion strategy variants, inclusion of clinical features, and depth of attention modules. Statistical significance levels are represented as follows: “*” indicates p<0.05, “**” indicates p<0.01, and “***” indicates p<0.001.

The analysis of attention depth identifies the area of the relationship between model capacity and the refinement of the representational hierarchy, particularly the area of diminishing returns of layer addition. Table 15 demonstrates that a single attention layer achieves 88.2 accuracy and 93.6 AUC, which proposes that contextual modeling is insufficient. With two layers, it has been observed that accuracy increases to 89.6 and AUC to 94.8; thus, deeper attention can improve the interaction and quality of the underlying representation. On the other hand, the peak performance is attained by three layers: 90.5 accuracy, 89.6 precision, 88.9 recall, 89.2 F1-score, and 95.8 AUC, indicating the best combination of model capacity and generalization. The addition of a fourth layer results in only a slight improvement: 90.4 accuracy and 95.7 AUC, indicating that the model has reached a state of saturation and minimal returns with further depth. A visual summary of the removal of components, fusions, contributions of clinical features, and attention depth is illustrated in Figure 8.

The ablation analysis is further expanded to describe the independent contributions of the attention module and the multimodal fusion module. Removing the attention module reduces the model’s ability to place attention on diagnostically relevant lesion regions. Removing this module results in lower sensitivity to complex, diagnostically relevant lesion classes. On the other hand, removing the fusion module results in the model’s limited ability to leverage complementary clinical metadata. Consequently, this reduces the model’s contextual reasoning and robustness across multiple datasets. Performance drop is not the same across all classes. The melanoma class, which is clinically relevant, shows a greater drop in sensitivity when either the attention component or the multimodal fusion component is removed. Removing both modules results in the largest drop in the Austral Melanoma Classification. The results of the ablation study confirm that the model’s components are relevant and necessary for achieving high performance across clinically relevant diagnostic classes.

The results in Table 16 show that the greatest melanoma class recall/sensitivity is achieved by the complete model. Thus, the complete model achieves higher sensitivity in detecting malignant lesions. Removing the attention component reduces melanoma sensitivity. This suggests that the attention component is required to detect different lesion characteristics. Finally, the average sensitivity was lowest when the fusion component was removed. This suggests that the metadata from clinical documents complements the contextual information. This component, combined with clinical contextual information, is likely to provide the needed information to detect melanoma. It is likely that the other model components are also required to achieve class-level performance in clinically relevant diagnostic classes, as this would have a negative impact. This ablation study, in conjunction with the other model fusion and attention components, serves multiple functions. The attention and fusion components serve both as clinically relevant diagnostic components and as means to enhance the model’s overall performance.

### 4.6. Computational Efficiency and Model Complexity

The trade-off analysis balances the model’s complexity and predictive capabilities, showing that the proposed model’s greater complexity achieved more accurate results without requiring additional computation. As is shown in Table 17, ResNet50 has 25.6 M parameters, 4.1 FLOPs, and infers in 12.5 ms with 89.3 AUC, whereas EfficientNet-B4 requires 19.3 M parameters and 4.5 FLOPs, inferring in 15.8 ms with 91.5 AUC. DenseNet121 achieves 90.4 AUC with 10.2 ms of inference, requiring 8.0 M parameters and 2.9 FLOPs. ViT-Base, on the other hand, has an AUC of 92.6 but with a lot more computation: 86.0 M parameters, 17.6 FLOPs, and 28.4 ms of inference. The CNN + Attention model achieves an AUC of 93.4 with 32.4 M parameters and 6.8 FLOPs, requiring 18.6 ms. The proposed multimodal model achieves an AUC of 95.8 with the most parameters (34.7 M) and 7.2 FLOPs, taking 19.3 ms of inference time, meaning the increase in computational load is negligible compared to CNN + Attention.

The efficiency analysis, broken down by dataset, shows that the model behaves computationally the same across datasets of different sizes and characteristics, meaning it can be deployed without worrying about performance changes. The results in Table 18 show tightly bounded inference times of 19.2 ms for ISIC 2019, 19.5 ms for ISIC 2020, and 19.1 ms for HAM10000, and the combined evaluation shows 19.3 ms, which means across different data distributions, the variation in ISIC 2019 data is of no significance. The model architecture is dataset-dependent, owing to dataset-specific complexities and computational costs, which remained constant across datasets at 7.2 FLOPs. Memory consumption across the datasets ranged from 510 MB (HAM10000) to 514 MB (ISIC 2020), with a maximum of 4 MB, consistent with the combined setting (513 MB), indicating no dataset-dependent variance.

The trade-offs between performance and efficiency demonstrate that the new model offers greater predictive power with limited computing power and effective resource efficiency, while compromising predictive power. The data presented in Table 19 show that ResNet50 reports an AUC of 89.3 at 25.6 parameters, 4.1 FLOPs, and 12.5 ms of inference, and that EfficientNet-B4 shows an improvement in AUC of 91.5 at 19.3 parameters and 4.5 FLOPs, but at 15.8 ms. DenseNet121 shows 8.0 lightweight parameters, 2.9 FLOPs, and an AUC of 90.4 at a latency of 10.2 ms. In comparison, ViT-Base achieves 92.6 AUC at a cost of 86.0 parameters and 17.6 FLOPs, with a much higher inference time of 28.4 ms. At 93.4 AUC, with 32.4 parameters and 6.8 FLOPs, and an inference ms latency of 18.6 ms, the CNN + Attention model offers the best trade-off between efficiency and performance. At 95.8 AUC, the proposed multimodal model achieves the best overall performance, with a cost of 34.7 parameters, 7.2 FLOPs, and 19.3 ms of inference time. Compared to the CNN + Attention model, the proposed model achieves a 3.2 AUC gain over ViT-Base while requiring much lower computational resources than transformer models.

### 4.7. Statistical Significance Analysis

The performance improvements are not the result of random chance; instead, they are reliable and repeatable across evaluations. Table 20 shows that Wilcoxon’s signed rank test also supports improvements and shows that there are also improvements ViT. Regarding accuracy, the *p*-value is 0.002. In precision, 0.003, 0.002 for recall, 0.002 for the F1 score, and 0.001 for AUC. Improvements are also seen in the non-parametric setting when compared with the CNN + Attention model. Consistency is also evident in the low *p*-values of 0.004 for accuracy, 0.005 for precision, 0.004 for recall, 0.004 for F1 score, and 0.003 for AUC. Downstream tasks show robustness in the hybrid baselines. Bootstrap testing shows the best baseline low *p*-value of 0.001, with AUC also showing improvements at *p* = 0.001, reinforcing stability across the high-resampled distribution.

The statistical breakdown by class shows that the same improvements in the proposed model are observed across all lesion categories, regardless of clinical significance or data distribution. Table 21 shows a high level of significance for melanoma with an almost 0.001 *p*-value for the *t*-test, 0.002 for the Wilcoxon, and <0.001 for the bootstrap, thus demonstrating strong confidence in the ability of the model to identify malignant cases. Improvements are also seen for nevus and benign keratosis, where the *t*-test value drops below 0.001, and the Wilcoxon value is 0.003. This confirms the dominant improvement in these lesions. Basal cell carcinoma also shows strong significance, with a Wilcoxon *p*-value of 0.002 and a bootstrap *p*-value of <0.001, indicating consistent improvement across all malignant lesions. Actinic keratosis has a *t*-test value of 0.001 and a Wilcoxon value of 0.004, both of which are quite high, indicating strong consistency and remaining quite high. This is due to the variability in the lesions. The same is true for Dermatofibroma, with a *t*-test value of 0.002 and a Wilcoxon value of 0.005. This shows a moderate variability and consistent improvement. Also, Vascular lesions show a lot of consistency with high significance (<0.001) *t*-test and (<0.003) Wilcoxon tests.

The proposed model maintains consistent performance improvements, as statistically validated, even across various domain shifts, as demonstrated by cross-dataset evaluation. All transfer configurations shown in Table 22 have *t*-test values <0.001, demonstrating that these performance improvements are statistically significant. For ISIC 2019 to ISIC 2020, a Wilcoxon value of 0.003 and a bootstrap value <0.001 indicate that these values remain consistent with significant distributional differences. The reverse transfer from ISIC 2020 to ISIC 2019 shows a Wilcoxon value of 0.002, demonstrating consistent bidirectional generalization. Also, the transfer from HAM10000 to ISIC 2019 has a Wilcoxon value of 0.002 and a bootstrap value of <0.001, indicating that better performance is obtained when a larger dataset is used to train the model and a different distribution is used to evaluate it. The transfer from ISIC 2019 to HAM10000 shows significant dependence (*p*-value = 0.003), demonstrating stable adaptation of data across datasets with different class compositions. Finally, the sample with a Wilcoxon value of 0.003 and a *t*-test value of <0.001 shows that these improvements are not sample-specific, and are consistent across many domain changes.

The analysis of the effect size demonstrates the magnitude of improvement achieved by the proposed model. In this case, the improvement is noteworthy statistically and practically. When compared to ResNet50, as shown in Table 23, the model has a Cohen’s d of 1.85 and a Cliff’s delta of 0.78, demonstrating a significant gap in the separated distributions of performance. Compared to EfficientNet-B4, the model has a Cohen’s d of 1.62 and a Cliff’s delta of 0.74, demonstrating consistent improvement over efficient convolutional architectures. In comparison to ViT, the model has a Cohen’s d of 1.48 and a Cliff’s delta of 0.70, further demonstrating that the proposed model is better than the ViT transformer-based models by a significant amount. Likewise, the comparison to CNN + Attention shows a Cohen’s d of 1.32 and a Cliff’s delta of 0.66, indicating that even very strong hybrid models are still significantly outperformed by CNN + Attention. The best baseline comparative analysis yields a Cohen’s d of 1.41 and a Cliff’s delta of 0.69, indicating that across all measures, the model shows consistent, significant improvement.

To offer a more detailed statistical analysis, several key evaluation metrics are provided with their respective confidence intervals. As such, 95% confidence intervals are generated from several repeated experiments, capturing the model’s performance variability. For a certain metric μ, confidence intervals are formulated as follows over *n* independent trials having a standard deviation σ, where *n* is the number of runs, and μ is the said metric:

(43) $$\mu \pm 1.96\cdot \frac{\sigma }{\sqrt{n}}$$

Point estimates are static, but incorporating confidence intervals offers a dynamic depiction of performance stability. Statistical versus practical significance illustrates improvements on a continuum of, respectively, uncommon versus real-world implications. Improvements in AUC, recall, and other metrics in skin cancer diagnosis yield fewer errors of judgment and promote early identification of malignant lesions. Over baseline models, AUC and F1-score improvements suggest improvements in discrimination and performance integrity, both of which are clinically important. This is especially true in the context of early melanoma detection, where small improvements are clinically impactful. To further improve the proposed model, effect sizes are considered alongside *p*-values. Frequent, positive results across datasets yield a sustained moderate-to-strong effect size. Clinically, enhanced Diagnostics and AUC help identify malignant lesions and provide solid evidence to reduce diagnostic errors in these cases. This potential confirms the proposed model’s combined utility in practical clinical settings.

### 4.8. Ethical Considerations

The current study uses fully anonymized dermoscopy datasets, ensuring the study neither processes any identifiable information nor interacts with human subjects. The study complies with routine ethical principles of secondary data analysis. The ethical implications of AI-powered diagnostic systems, particularly their fairness, accountability, and transparency, are critically examined. The previously discussed bias in datasets, specifically the bias in visual element color diversity, directly impacts the fairness of model performance across varying populations, thereby advocating for equitable models and bias-aware evaluations.

The proposed framework aims to serve as a clinical decision support system, not as a substitute for a medical doctor. The final assessment of a patient’s diagnosis should always be in the hands of practitioners. AI, in these circumstances, should be used as a technology to assist the practitioners in improving the diagnostic process. Bias and transparency of models, data privacy, and evidence of compliance with clinical frameworks and regulations should be prioritized without exception before the technology is incorporated in the assistive systems of diagnostics. Explainability and prospective clinical research validation should be the priority in the proposed framework.

### 4.9. Discussion

The experimental results support the claim that combining multiple data sources yields statistically outperforms baseline models in image classification performance for skin cancer. The improvements observed across the ISIC 2019, ISIC 2020, and HAM10000 datasets demonstrate that adding clinical metadata to visual data enables the model to better understand and exploit patterns that emerge only when the images are combined with the metadata. This is also reflected in improvements in AUC and F1 scores, which assess class discrimination and class decision-making in a balanced way. The generalization results across datasets demonstrate the broader improvements offered by the proposed methods. The results from unimodal approaches capture and describe data features. This allows the model to describe and capture less clinical data without affecting its performance, which is a detrimental factor when deployed in clinical settings, where data and devices collect and describe data in different ways. The lower data and devices capture and describe the data differently. The lower performance loss seen in transfer settings indicates that the model’s weights are learned from its less salient visual features and are less dominated by them than the model’s weights. The model’s clinical reliability has been assessed by analyzing each class. In medical settings, the reduced risk of missed critical diagnoses is supported by improved recall of malignant melanoma, which poses the greatest risk. Maintaining high precision helps control false positives and avoid unnecessary clinical actions. Balanced performance across rare and common classes indicates that the model successfully classifies imbalanced data, a common issue in medical imaging datasets. Among all model components, the multimodal fusion mechanism is the most important, and accurate modality interactions have a significant positive effect on model performance. The encoder, based on attention mechanisms, improves feature representation, and integrating clinical features enhances model performance by providing a more accurate context for model reasoning. The overall framework’s performance reinforces the benefits of these elements by showing a consistent decline when any of them is absent.

The framework aims for clinical application, providing outputs that are simple and easy to use. The predictive module aims to operate as a decision support system, analytically interpreting dermoscopic images while simultaneously providing probability-based predictions. With the addition of clinical metadata, such as relevant patient characteristics and lesion location, the predictive module’s probabilities will be more relevant to everyday clinical workflow and easier to interpret. In the clinical setting, the framework will serve as an intra-dermoscopy aid or a hospital diagnostic information system module. This module will be able to interpret patients’ clinical data in real time and assist with diagnostic-suggestive predictions. This will aid the system in performing to enhance dermatoscopic screening and triaging. The system can also serve as a diagnostic dual-control request in situations where there is a shortage of medical professionals or in resource-constrained settings.

In melanoma detection, false negatives incur a greater cost of misclassification than false positives. Because of this, the framework’s ability to screen melanoma cases in real-world datasets is further validated by the high sensitivities for melanoma detection across datasets. Fortifying specificity in this framework will lead to fewer diagnoses, fewer wasteful and non-beneficial clinical interventions, and less psychosocial stress. Strongly imbalanced optimization of sensitivity and specificity within the framework will enable clinicians to derive accurate, meaningful clinical diagnoses, further establishing the framework’s utility as a highly valuable clinical decision support system.

The proposed model shows a good balance between efficiency and performance. While attention techniques and multimodality do involve additional complexity compared to lightweight CNNs, they are still considerably less complex than transformer-based models. Considering the fixed inference time and a moderate number of parameters, the potential for assimilating the model into clinical workflows is reasonable. The thorough analysis of statistical significance justifies the confidence in the observed improvements, and the improvements are not just a result of chance. In the context of the numerous experiments, the combination of small *p*-values and considerable effect sizes demonstrates the performance improvements in both practical and statistical aspects. This evidence further illustrates the effectiveness and promise the proposed model holds in practical applications.

An important challenge in deploying AI-based dermatological systems is the datasets used to train machine learning models. Most public datasets of dermatological images are biased toward lighter skin tones. Examples of those datasets used in this study are ISIC and HAM10000. If a training dataset is supplemented with more diverse skin types, the model is more likely not to generalize to the population of interest, particularly the underrepresented population. The model is more likely to underrepresent the population with darker skin tones that require specialized features to be discerned. For this reason, the model is most likely biased in terms of representation and fairness, and it is likely to be unreliable in real-world clinical settings. More diverse, representative datasets that include populations with darker skin tones, and more robust evaluation of systems that model populations with more diverse darker skin tones, are important ways to address this problem.

Real-world deployment is analyzed, acknowledging limitations despite promising performance. First, the use of clinical metadata means the model is incomplete and inconsistent across healthcare systems. Second, domain shifts may introduce variety despite cross-dataset generalization. Third, the current framework is tested on publicly available datasets that do not accurately reflect the clinical population. There is no way to assess the model’s real-world performance or user interaction, as there has been no validation. Lastly, the model outputs probabilities, but clinical reasoning and justification are beyond the scope of the features.

## 5. Conclusions

This research introduces an innovative multimodal deep learning framework that integrates dermoscopic imaging capabilities with clinical information to support skin cancer diagnosis. The proposed method’s framework fuses attention-based feature encoding with a novel means to capture cross-modality and inter-modality complementary features. The most recent data shows improvements using the proposed method and framework across the three benchmarks, including ISIC 2019, ISIC 2020, and HAM10000. The proposed method and framework improve ISIC 2020 performance over ISIC 2019 and HAM10000 across all ISIC 2020 lesions, achieving higher specificity. The proposed method and framework improve AUC and F1 scores and explain lesions better than previous methods. The framework improves the model’s performance by explaining ISIC lesions and evaluating advanced melanoma lesions using the proposed method, thereby strongly reducing the likelihood of clinical melanoma lesions being misdiagnosed. Traditional ISIC benchmarks evaluate the model’s non-specialized properties and ISIC specialized content; the ISIC numbers meaningfully and statistically explain the benchmarks. Each framework and method component improves the detection of ISIC lesions. The method yields high performance and is well-balanced and specialized. The remaining studies on the proposed method will evaluate the framework’s specialized properties and propose clinical settings. Future endeavors must prioritize prospective clinical validation and seamless integration with electronic health record systems. Additionally, improved clinician trust and adoption require the development of more transparent mechanisms for explainability. Subsequent research will also address the implementation of skin tone annotations, stratified evaluations, and fairness-centric learning approaches to guarantee the same level of diagnostic equity for all patients.

## Figures and Tables

**Table 1:** Comparison of recent state-of-the-art methods across HAM10000, ISIC 2020, and ISIC 2019 datasets.

Method	Dataset	Task	Accuracy (%)	F1 Score	AUC (%)
Hybrid CNN-Transformer (KAN) [18]	HAM10000	7-class	92.81	92.47	–
QANA [19]	HAM10000	7-class	91.60	91.00	93.40
EfficientNetV2-L [20]	HAM10000	7-class	91.15	85.45	99.33
Multi-task ABCDE [21]	HAM10000	7-class	89.00	–	96.00
SLIMP [22]	HAM10000	7-class	85.90	65.00	90.10
ASFF-ResNet50 [27]	ISIC 2020	Binary	93.18	–	97.17
DermFM-Zero [26]	ISIC 2020	Binary	–	95.30	87.20
DermLIP [26]	ISIC 2020	Binary	–	76.20	82.40
MAKE [23]	ISIC 2020	Binary	–	76.60	81.30
BiomedCLIP [24]	ISIC 2020	Binary	–	97.00	72.90
CoPA [28]	ISIC 2019	Multi-class	90.10	89.40	95.20
SLIMP [22]	ISIC 2019	Multi-class	88.70	87.90	94.60
IUAV-IP [25]	ISIC 2019	Multi-class	87.50	–	93.10
DermFM-Zero [26]	ISIC 2019	Multi-class	–	86.20	92.40
MAKE [23]	ISIC 2019	Multi-class	–	87.10	91.80

**Table 2:** Training hyperparameters and configuration.

Hyperparameter	Value
Input Resolution	224×224
Batch Size	32
Initial Learning Rate	$1\times 10^{-4}$
Optimizer	Adam
Weight Decay	$1\times 10^{-5}$
Dropout Rate	0.5
Number of Epochs	100
Early Stopping Patience	15
LR Scheduler	Cosine Decay
Horizontal Flip Prob.	0.5
Rotation Range	±20°
Color Jitter Strength	0.2
Loss Function	Cross-Entropy

**Table 3:** Quantitative performance comparison across datasets.

Method	Accuracy	Precision	Recall	F1-Score	AUC
ISIC 2019					
ResNet50	83.2±0.8	81.5±0.9	80.9±1.0	81.2±0.9	89.3±0.7
EfficientNet-B4	85.6±0.7	84.2±0.8	83.5±0.9	83.8±0.8	91.5±0.6
ViT	86.8±0.6	85.7±0.7	84.9±0.8	85.3±0.7	92.6±0.5
CNN + Attention	87.9±0.6	86.8±0.7	86.0±0.7	86.4±0.7	93.4±0.5
Multimodal (Proposed)	90.5±0.5	89.6±0.6	88.9±0.6	89.2±0.6	95.8±0.4
ISIC 2020					
ResNet50	81.4±0.9	79.8±1.0	78.9±1.1	79.3±1.0	87.6±0.8
EfficientNet-B4	83.7±0.8	82.4±0.9	81.6±0.9	82.0±0.9	89.9±0.7
ViT	84.9±0.7	83.8±0.8	83.0±0.8	83.4±0.8	91.0±0.6
CNN + Attention	86.1±0.7	85.0±0.7	84.2±0.8	84.6±0.7	92.2±0.6
Multimodal (Proposed)	88.7±0.6	87.9±0.6	87.0±0.7	87.4±0.6	94.6±0.5
HAM10000					
ResNet50	84.5±0.8	83.2±0.9	82.5±0.9	82.8±0.9	90.2±0.7
EfficientNet-B4	86.9±0.7	85.6±0.8	84.8±0.8	85.2±0.8	92.1±0.6
ViT	88.1±0.6	87.0±0.7	86.2±0.7	86.6±0.7	93.3±0.5
CNN + Attention	89.3±0.6	88.2±0.6	87.5±0.7	87.8±0.6	94.1±0.5
Multimodal (Proposed)	91.8±0.5	90.9±0.5	90.1±0.6	90.5±0.5	96.3±0.4

**Table 4:** Dataset-wise performance summary of proposed model.

Dataset	Accuracy	Precision	Recall	F1-Score	AUC
ISIC 2019	90.5±0.5	89.6±0.6	88.9±0.6	89.2±0.6	95.8±0.4
ISIC 2020	88.7±0.6	87.9±0.6	87.0±0.7	87.4±0.6	94.6±0.5
HAM10000	91.8±0.5	90.9±0.5	90.1±0.6	90.5±0.5	96.3±0.4
Combined	90.9±0.5	90.0±0.5	89.3±0.6	89.6±0.5	95.9±0.4

**Table 5:** Class-wise performance on ISIC 2019.

Class	Accuracy	Precision	Recall	F1-Score	AUC
Melanoma	88.6±0.7	87.9±0.8	86.8±0.9	87.3±0.8	94.5±0.6
Nevus	92.4±0.5	91.6±0.6	91.0±0.6	91.3±0.6	96.8±0.4
Basal Cell Carcinoma	90.2±0.6	89.4±0.7	88.6±0.7	89.0±0.7	95.7±0.5
Actinic Keratosis	87.9±0.7	87.0±0.8	86.1±0.9	86.5±0.8	94.1±0.6
Benign Keratosis	91.1±0.6	90.3±0.7	89.5±0.7	89.9±0.7	95.9±0.5
Dermatofibroma	89.3±0.7	88.5±0.8	87.6±0.8	88.0±0.8	94.8±0.6
Vascular Lesion	90.7±0.6	89.9±0.7	89.1±0.7	89.5±0.7	95.6±0.5
Squamous Cell Carcinoma	88.8±0.7	88.0±0.7	87.2±0.8	87.6±0.7	94.9±0.6

**Table 6:** Class-wise performance on ISIC 2020.

Class	Accuracy	Precision	Recall	F1-Score	AUC
Melanoma	87.2±0.8	86.5±0.8	85.6±0.9	86.0±0.8	93.9±0.6
Benign	90.1±0.6	89.3±0.7	88.5±0.7	88.9±0.7	95.2±0.5

**Table 7:** Class-wise performance on HAM10000.

Class	Accuracy	Precision	Recall	F1-Score	AUC
Melanoma	89.5±0.6	88.7±0.7	87.9±0.7	88.3±0.7	95.6±0.5
Nevus	93.1±0.5	92.3±0.5	91.6±0.6	91.9±0.5	97.2±0.4
Basal Cell Carcinoma	91.3±0.6	90.5±0.6	89.7±0.7	90.1±0.6	96.4±0.5
Actinic Keratosis	88.8±0.7	88.0±0.7	87.2±0.8	87.6±0.7	94.9±0.6
Benign Keratosis	92.0±0.5	91.2±0.6	90.4±0.6	90.8±0.6	96.6±0.4
Dermatofibroma	90.2±0.6	89.4±0.7	88.6±0.7	89.0±0.7	95.7±0.5
Vascular Lesion	91.6±0.5	90.8±0.6	90.0±0.6	90.4±0.6	96.3±0.4

**Table 8:** Cross-dataset generalization performance.

Method	Accuracy	Precision	Recall	F1-Score	AUC
ISIC 2019 → ISIC 2020					
ResNet50	75.2±1.0	73.8±1.1	72.9±1.2	73.3±1.1	82.4±0.9
EfficientNet-B4	77.6±0.9	76.3±1.0	75.4±1.0	75.8±1.0	84.6±0.8
ViT	78.9±0.8	77.8±0.9	76.9±0.9	77.3±0.9	85.9±0.7
CNN + Attention	80.1±0.8	79.0±0.8	78.2±0.9	78.6±0.8	87.2±0.7
Multimodal (Proposed)	83.4±0.7	82.5±0.7	81.6±0.8	82.0±0.7	90.1±0.6
ISIC 2020 → ISIC 2019					
ResNet50	76.1±1.0	74.7±1.1	73.8±1.1	74.2±1.1	83.1±0.9
EfficientNet-B4	78.5±0.9	77.2±0.9	76.3±1.0	76.7±0.9	85.3±0.8
ViT	79.8±0.8	78.7±0.9	77.9±0.9	78.3±0.9	86.6±0.7
CNN + Attention	81.0±0.8	79.9±0.8	79.1±0.9	79.5±0.8	87.8±0.7
Multimodal (Proposed)	84.2±0.7	83.3±0.7	82.5±0.8	82.9±0.7	90.8±0.6
HAM10000 → ISIC 2019					
ResNet50	77.4±0.9	76.0±1.0	75.2±1.0	75.6±1.0	84.2±0.8
EfficientNet-B4	79.6±0.8	78.4±0.9	77.5±0.9	77.9±0.9	86.1±0.7
ViT	80.9±0.8	79.8±0.8	78.9±0.9	79.3±0.8	87.5±0.7
CNN + Attention	82.2±0.7	81.1±0.8	80.3±0.8	80.7±0.8	88.9±0.6
Multimodal (Proposed)	85.6±0.6	84.7±0.7	83.9±0.7	84.3±0.7	91.7±0.5
ISIC 2019 → HAM10000					
ResNet50	76.8±1.0	75.5±1.0	74.6±1.1	75.0±1.0	83.7±0.9
EfficientNet-B4	79.1±0.9	77.9±0.9	77.0±1.0	77.4±0.9	85.6±0.8
ViT	80.4±0.8	79.3±0.9	78.5±0.9	78.9±0.9	86.9±0.7
CNN + Attention	81.6±0.8	80.6±0.8	79.7±0.9	80.1±0.8	88.2±0.7
Multimodal (Proposed)	84.9±0.7	84.0±0.7	83.1±0.8	83.5±0.7	91.0±0.6

**Table 9:** Aggregated cross-dataset performance of proposed model.

Train → Test	Accuracy	Precision	Recall	F1-Score	AUC
ISIC 2019 → ISIC 2020	83.4±0.7	82.5±0.7	81.6±0.8	82.0±0.7	90.1±0.6
ISIC 2020 → ISIC 2019	84.2±0.7	83.3±0.7	82.5±0.8	82.9±0.7	90.8±0.6
HAM10000 → ISIC 2019	85.6±0.6	84.7±0.7	83.9±0.7	84.3±0.7	91.7±0.5
ISIC 2019 → HAM10000	84.9±0.7	84.0±0.7	83.1±0.8	83.5±0.7	91.0±0.6
Average	84.5±0.7	83.6±0.7	82.8±0.8	83.2±0.7	90.9±0.6

**Table 10:** Component-wise ablation on ISIC 2019.

Variant	Accuracy	Precision	Recall	F1-Score	AUC
Full Model	90.5±0.5	89.6±0.6	88.9±0.6	89.2±0.6	95.8±0.4
w/o Attention Encoder	88.2±0.7	87.3±0.7	86.5±0.8	86.9±0.7	93.6±0.5
w/o Fusion Module	86.4±0.8	85.6±0.8	84.7±0.9	85.1±0.8	92.1±0.6
w/o Clinical Features	87.1±0.7	86.3±0.8	85.5±0.8	85.9±0.8	92.9±0.6
CNN Only	85.2±0.9	84.4±0.9	83.6±1.0	84.0±0.9	90.8±0.7

**Table 11:** Component-wise ablation on ISIC 2020.

Variant	Accuracy	Precision	Recall	F1-Score	AUC
Full Model	88.7±0.6	87.9±0.6	87.0±0.7	87.4±0.6	94.6±0.5
w/o Attention Encoder	86.3±0.7	85.5±0.7	84.7±0.8	85.1±0.7	92.4±0.6
w/o Fusion Module	84.8±0.8	84.0±0.8	83.2±0.9	83.6±0.8	91.0±0.6
w/o Clinical Features	85.5±0.7	84.7±0.7	83.9±0.8	84.3±0.7	91.7±0.6
CNN Only	83.7±0.9	82.9±0.9	82.1±1.0	82.5±0.9	89.8±0.7

**Table 12:** Component-wise ablation on HAM10000.

Variant	Accuracy	Precision	Recall	F1-Score	AUC
Full Model	91.8±0.5	90.9±0.5	90.1±0.6	90.5±0.5	96.3±0.4
w/o Attention Encoder	89.6±0.6	88.7±0.6	87.9±0.7	88.3±0.6	94.2±0.5
w/o Fusion Module	87.9±0.7	87.0±0.7	86.2±0.8	86.6±0.7	92.8±0.6
w/o Clinical Features	88.5±0.7	87.7±0.7	86.9±0.8	87.3±0.7	93.5±0.5
CNN Only	86.8±0.8	85.9±0.8	85.1±0.9	85.5±0.8	91.6±0.6

**Table 13:** Fusion strategy ablation.

Fusion Method	Accuracy	Precision	Recall	F1-Score	AUC
Concatenation	88.1±0.7	87.2±0.7	86.4±0.8	86.8±0.7	93.2±0.6
Element-wise Sum	87.5±0.8	86.6±0.8	85.8±0.9	86.2±0.8	92.7±0.6
Gated Fusion	89.2±0.6	88.3±0.6	87.5±0.7	87.9±0.6	94.1±0.5
Attention Fusion (Proposed)	90.5±0.5	89.6±0.6	88.9±0.6	89.2±0.6	95.8±0.4

**Table 14:** Clinical feature contribution analysis.

Input Modality	Accuracy	Precision	Recall	F1-Score	AUC
Image Only	87.1±0.7	86.3±0.8	85.5±0.8	85.9±0.8	92.9±0.6
Clinical Only	78.4±1.0	77.6±1.1	76.8±1.1	77.2±1.1	85.3±0.9
Image + Clinical (Concat)	88.4±0.6	87.6±0.7	86.8±0.7	87.2±0.7	93.8±0.5
Image + Clinical (Proposed)	90.5±0.5	89.6±0.6	88.9±0.6	89.2±0.6	95.8±0.4

**Table 15:** Attention depth analysis.

Attention Layers	Accuracy	Precision	Recall	F1-Score	AUC
1 Layer	88.2±0.7	87.3±0.7	86.5±0.8	86.9±0.7	93.6±0.5
2 Layers	89.6±0.6	88.7±0.6	87.9±0.7	88.3±0.6	94.8±0.5
3 Layers	90.5±0.5	89.6±0.6	88.9±0.6	89.2±0.6	95.8±0.4
4 Layers	90.4±0.5	89.5±0.6	88.8±0.6	89.1±0.6	95.7±0.4

**Table 16:** Class-wise ablation analysis for clinically important melanoma detection.

Model Variant	Accuracy	Precision	Recall/Sensitivity	AUC
Proposed Full Model	88.6	87.9	86.8	94.5
Without Attention Module	86.1	85.2	83.4	91.7
Without Fusion Module	85.7	84.9	82.8	91.2
Image-only Variant	84.9	84.1	81.9	90.6
Metadata-only Variant	76.5	75.8	73.6	82.4

**Table 17:** Computational complexity and efficiency comparison.

Method	Parameters (M)	FLOPs (G)	Inference Time (ms)	AUC
ResNet50	25.6±0.2	4.1±0.1	12.5±0.5	89.3±0.7
EfficientNet-B4	19.3±0.2	4.5±0.1	15.8±0.6	91.5±0.6
DenseNet121	8.0±0.1	2.9±0.1	10.2±0.4	90.4±0.7
ViT-Base	86.0±0.3	17.6±0.2	28.4±0.8	92.6±0.5
CNN + Attention	32.4±0.3	6.8±0.2	18.6±0.6	93.4±0.5
Multimodal (Proposed)	34.7±0.3	7.2±0.2	19.3±0.6	95.8±0.4

**Table 18:** Dataset-wise inference efficiency of proposed model.

Dataset	Inference Time (ms)	FLOPs (G)	Memory (MB)
ISIC 2019	19.2±0.6	7.2±0.2	512±10
ISIC 2020	19.5±0.6	7.2±0.2	514±10
HAM10000	19.1±0.5	7.2±0.2	510±9
Combined	19.3±0.6	7.2±0.2	513±10

**Table 19:** Performance–efficiency trade-off analysis.

Method	AUC	Parameters (M)	FLOPs (G)	Inference Time (ms)
ResNet50	89.3±0.7	25.6±0.2	4.1±0.1	12.5±0.5
EfficientNet-B4	91.5±0.6	19.3±0.2	4.5±0.1	15.8±0.6
DenseNet121	90.4±0.7	8.0±0.1	2.9±0.1	10.2±0.4
ViT-Base	92.6±0.5	86.0±0.3	17.6±0.2	28.4±0.8
CNN + Attention	93.4±0.5	32.4±0.3	6.8±0.2	18.6±0.6
Multimodal (Proposed)	95.8±0.4	34.7±0.3	7.2±0.2	19.3±0.6

**Table 20:** Statistical significance tests across models.

Test	Comparison	Accuracy	Precision	Recall	F1-Score	AUC
Paired *t*-test	Proposed vs. ResNet50	<0.001	<0.001	<0.001	<0.001	<0.001
Paired *t*-test	Proposed vs. EfficientNet-B4	<0.001	<0.001	<0.001	<0.001	<0.001
Wilcoxon Signed-Rank	Proposed vs. ViT	0.002±0.001	0.003±0.001	0.002±0.001	0.002±0.001	0.001±0.001
Wilcoxon Signed-Rank	Proposed vs. CNN + Attention	0.004±0.001	0.005±0.001	0.004±0.001	0.004±0.001	0.003±0.001
Bootstrap Test	Proposed vs. Best Baseline	0.001±0.0005	0.001±0.0005	0.001±0.0005	0.001±0.0005	<0.001

**Table 21:** Class-wise statistical significance (ISIC 2019).

Class	*t*-Test (*p*-Value)	Wilcoxon	Bootstrap	Significance
Melanoma	<0.001	0.002±0.001	<0.001	Significant
Nevus	<0.001	0.003±0.001	<0.001	Significant
Basal Cell Carcinoma	<0.001	0.002±0.001	<0.001	Significant
Actinic Keratosis	0.001±0.001	0.004±0.001	0.001±0.0005	Significant
Benign Keratosis	<0.001	0.003±0.001	<0.001	Significant
Dermatofibroma	0.002±0.001	0.005±0.001	0.001±0.0005	Significant
Vascular Lesion	<0.001	0.003±0.001	<0.001	Significant

**Table 22:** Cross-dataset statistical significance.

Transfer Setting	*t*-Test	Wilcoxon	Bootstrap	Significance
ISIC 2019 → ISIC 2020	<0.001	0.003±0.001	<0.001	Significant
ISIC 2020 → ISIC 2019	<0.001	0.002±0.001	<0.001	Significant
HAM10000 → ISIC 2019	<0.001	0.002±0.001	<0.001	Significant
ISIC 2019 → HAM10000	<0.001	0.003±0.001	<0.001	Significant
Average	<0.001	0.003±0.001	<0.001	Significant

**Table 23:** Effect size analysis.

Comparison	Cohen’s d	Cliff’s Delta	Interpretation
Proposed vs. ResNet50	1.85±0.10	0.78±0.05	Large
Proposed vs. EfficientNet-B4	1.62±0.09	0.74±0.05	Large
Proposed vs. ViT	1.48±0.08	0.70±0.04	Large
Proposed vs. CNN + Attention	1.32±0.08	0.66±0.04	Large
Best Baseline vs. Proposed	1.41±0.09	0.69±0.04	Large

## Data Availability

The implementation of this work can be found at https://github.com/imashoodnasir/Explainability-Guided-Multimodal-for-Brain-Tumor-Segmentation (accessed on 8 May 2026).
